# Antiprion Activity of DB772 and Related Monothiophene- and Furan-Based Analogs in a Persistently Infected Ovine Microglia Culture System

**DOI:** 10.1128/AAC.00811-16

**Published:** 2016-08-22

**Authors:** Kelcey D. Dinkel, James B. Stanton, David W. Boykin, Chad E. Stephens, Sally A. Madsen-Bouterse, David A. Schneider

**Affiliations:** a Veterinary Microbiology and Pathology, Washington State University, Pullman, Washington, USA; b Department of Pathology, University of Georgia, Athens, Georgia, USA; c Department of Chemistry, Georgia State University, Atlanta, Georgia, USA; d Department of Chemistry and Physics, Augusta University, Augusta, Georgia, USA; e United States Department of Agriculture, Agricultural Research Service, Animal Disease Research Unit, Pullman, Washington, USA

## Abstract

The transmissible spongiform encephalopathies are fatal neurodegenerative disorders characterized by the misfolding of the native cellular prion protein (PrP^C^) into the accumulating, disease-associated isoform (PrP^Sc^). Despite extensive research into the inhibition of prion accumulation, no effective treatment exists. Previously, we demonstrated the inhibitory activity of DB772, a monocationic phenyl-furan-benzimidazole, against PrP^Sc^ accumulation in sheep microglial cells. In an effort to determine the effect of structural substitutions on the antiprion activity of DB772, we employed an *in vitro* strategy to survey a library of structurally related, monothiophene- and furan-based compounds for improved inhibitory activity. Eighty-nine compounds were screened at 1 μM for effects on cell viability and prion accumulation in a persistently infected ovine microglia culture system. Eleven compounds with activity equivalent to or higher than that of DB772 were identified as preliminary hit compounds. For the preliminary hits, cytotoxicities and antiprion activities were compared to calculate the tissue culture selectivity index. A structure-activity relationship (SAR) analysis was performed to determine molecular components contributing to antiprion activity. To investigate potential mechanisms of inhibition, effects on PrP^C^ and PrP^Sc^ were examined. While inhibition of total PrP^C^ was not observed, the results suggest that a potential target for inhibition at biologically relevant concentrations is through PrP^C^ misfolding to PrP^Sc^. Further, SAR analysis suggests that two structural elements were associated with micromolar antiprion activity. Taken together, the described data provide a foundation for deeper investigation into untested DB compounds and in the design of effective therapeutics.

## INTRODUCTION

Prions are unique proteinaceous, infectious agents that cause fatal neurodegenerative disorders collectively known as the transmissible spongiform encephalopathies (TSEs). TSEs include Creutzfeldt-Jakob disease in humans, chronic wasting disease in cervids, and scrapie in sheep and goats (1, 2). Prions are comprised of protease-resistant, disease-associated isoforms (PrP^Sc^) of the prion protein (3). Prion protein in its native cellular form (PrP^C^) is a glycoprotein encoded by the host's *PRNP* gene and is highly expressed in multiple cell types, including neurons, microglia, and certain cells of the immune system (1 – 3). Misfolding to PrP^Sc^ promotes self-templated replication and accumulation within the central nervous system that lead to slowly progressive neurological dysfunction and eventually death (1 – 3). Currently, the mechanisms underlying this conversion are incompletely defined, and treatments to prevent or cure disease do not exist (4).

Due to the invariable lethality of prion disease, identification of compounds that prevent misfolding, replication, or accumulation is a vital goal (4 – 6). Previous studies to screen candidate antiprion compounds have primarily employed rodent cell culture systems chronically infected with rodent-adapted prion strains (7 – 10). Numerous structurally diverse compounds have since been identified and include sulfonated dyes (e.g., Congo red) (11), sulfated polyanions (e.g., pentosan polysulfate) (12 – 15), 2-aminothiazoles (e.g., IND24) (16 – 19), and polyene antibiotics (e.g., amphotericin B) (20). Unfortunately, these compounds have not demonstrated acceptable activity and bioavailability in animal studies, and those tested in human clinical trials failed to have significant effects (e.g., quinacrine) (10, 21, 22). Recently, the importance of species-specific models for the testing of antiprion compounds has been highlighted (23).

In 2012, we discovered the antiprion activity of the novel compound DB772, which is 2-(2-benzimidazolyl)-5-[4-(2-imidazolino)phenyl] furan dihydrochloride (24). DB772 was originally synthesized as a part of a library of DB compounds that represent structural derivatives of the parent molecule, furamidine (DB075) (25 – 28). Structural derivatives of furamidine are of broad interest because of potent activity demonstrated against parasitic microorganisms (e.g., Trypanosoma brucei) (29) and for bovine viral diarrhea virus (BVDV) (25, 26, 30, 31). Previous studies have described the efficient inhibitory activity of multiple derivatives in animal models (30, 31), and pharmacokinetic analysis has revealed the ability of some of these derivatives to penetrate the central nervous system (29). Using sheep-derived scrapie prions and a culture system of sheep microglia—a natural host cell target of scrapie prions—we found that micromolar treatment with DB772 led to near-maximal antiprion activity and minimal effect on cell viability.

Several possibilities may be considered the mechanism of antiprion activity for DB772. The DNA binding activity of DB772 and related analogs (27, 28, 32 – 38) suggests a potential to alter protein expression through direct effects on gene transcription. Alternatively, DB772 may bind directly to PrP^C^, PrP^Sc^, or other cofactors to interfere with efficient prion replication. For example, the direct binding of luminescent conjugated polymers (LCPs) to PrP^Sc^ inhibited prion replication by increasing protease resistance and preventing fragmentation into infectious particles (39). Several furamidine analogs contain a thiophene, which is a structural motif present in the putative pharmacophore of LCP analogs. This suggests that DB772 may share a similar mechanism of inhibitory action through direct interaction with PrP^Sc^. The availability of the structural library of DB compounds provides a rational means for further investigation into the antiprion activity of DB772. Analysis of the structure-activity relationship (SAR) could determine a structural basis for inhibition, further providing an opportunity to identify other DB compounds with improved inhibitory activity and a means by which to elucidate their mechanism of action.

## MATERIALS AND METHODS

A summary flow chart of the experimental *in vitro* strategy used to screen the DB compound library is provided as Fig. S1 in the supplemental material. In brief, DB compounds were tested using a previously described ovine microglial cell line (40) and were first screened for cytotoxicity and relative antiprion activity at 1 μM. Compounds with micromolar antiprion activities equivalent to or higher than that of DB772 were selected as preliminary hit compounds. Concentration-effect curves for each preliminary hit compound were subsequently produced in order to determine the tissue culture selectivity index (SI). All data were used to analyze the structure-activity relationship of DB compounds for antiprion activity. Furthermore, DB compounds were selected to determine the effects on PrP^C^ and PrP^Sc^, targets potentially relevant to the mechanism of antiprion activity.

### Ethics statement.

The Institutional Animal Care and Use Committees of Washington State University and the University of Washington approved all animal study protocols prior to initiation (permit numbers 4267, 4575, and 2610). The transgenic tg338 mice were kindly provided by Hubert Laude (Institut National de la Recherche Agronomique, France) (41 – 43).

### DB library of compounds.

The chemical library of compounds including DB772 was synthesized in the laboratories of two of the authors (D.W.B. and C.E.S.). A total of 89 compounds (see Table S1 in the supplemental material) were dissolved in sterile type I ultrapure water or dimethyl sulfoxide (DMSO) to a final stock concentration of 1, 5, or 10 mM. Stock solutions were aliquoted and stored at −20°C until use. Working concentrations were achieved by dilution of stock solutions into cell culture medium, sterile ultrapure water, or DMSO. No precipitate was visible following preparation of the most concentrated stock solutions or after dilution to micromolar working concentrations, suggesting that these DB compounds were water soluble.

### Cell lines.

For these experiments, persistently PrP^Sc^-positive and -negative cell lines of human telomerase-immortalized (hTERT) ovine microglia were utilized. As previously described (40), the persistently positive cells were originally inoculated with brain homogenate from sheep infected with classical scrapie, whereas the negative cell lines were originally inoculated with brain homogenate from a normal sheep. Cell line cultures were maintained at 37°C in OMEM (Opti-MEM supplemented with 10% heat-inactivated fetal bovine serum, 2 mM l-glutamine, 10 IU of penicillin, and 10 mg/ml of streptomycin) and passaged every 5 days. For passage, cells were lifted with 1× trypsin-EDTA in 1× phosphate-buffered saline (PBS; Gibco) and split 1/5 in 25-cm^2^ culture flasks. Prior to and throughout DB compound screening, cell line cultures were confirmed as persistently positive or negative for PrP^Sc^ by the standard scrapie cell assay (described below). The scrapie-infected cell line remained persistently PrP^Sc^ positive for over 100 passages during the course of this study.

### Cell viability.

The WST-1 assay was used to measure the 1 μM effect of each DB compound on cell viability. Briefly, PrP^Sc^-positive cells were passed from 25-cm^2^ culture flasks into a flat-bottomed, 96-well plate at a sufficient density (approximately 1,000 cells/well) to sustain log growth throughout the period of treatment. Following 24 h of incubation, well medium was exchanged with OMEM containing either 1 μM DB compound or 0.1% DMSO (vehicle control). After a 96-hour treatment, cell viability in each well was determined according to the manufacturer's directions by addition of 2-(4-iodophenyl)-3-(4-nitrophenyl)-5-(2,4-disulfophenyl)-2H-tetrazolium (WST-1; Roche Applied Science, USA). At 4 h of WST-1 incubation, optical density (OD) was measured at 450 and 620 nm using a microplate reader (SpectraMax 190; Molecular Devices). Cell viability was expressed as the percentage of absorbance (OD_450-620_) in wells containing DB compound-treated cells relative to those containing vehicle control-treated cells. Compounds were assayed in six replicates per plate. Thus, in order to adapt the screening of all DB library compounds to the 96-well plate format, plates contained a subset of compounds, DB772 (as an interassay standard), and the vehicle control (negative control). Each assay group was run in a minimum of three independent trials. The effects of treatments on percent viability were tested using a two-way analysis of variance (ANOVA) (PROC GLM; SAS for Windows, version 9.3; SAS Institute Inc., Cary, NC, USA) for the fixed effects of DB compound (treatment factor) and assay group (blocking factor). Dunnett's multiple-comparison test was used *post hoc* to compare the least-squares mean effect of each compound with the vehicle control (*P*_Dun_ < 0.05).

The WST-1 assay was similarly used to estimate the DB compound concentration that reduces percent viability to 50% (i.e., the 50% cytotoxic concentration [CC_50_]). This was done using a 1/4-log dilution series ranging from 10 μM to 0.1 μM (DMSO working concentration was either 0.1% or 0.316%). The CC_50_ was estimated from the general logistic function fit to concentration-response data by nonlinear regression (SigmaPlot ver. 13). The mean CC_50_ was determined from a minimum of three independent trials.

### Cell-based antiprion activity.

Antiprion activity was evaluated using the standard scrapie cell assay (SSCA) as adapted to sheep microglia (44). PrP^Sc^-positive cells were passed from 25-cm^2^ culture flasks into 12-well tissue culture plates at a density of approximately 30,000 cells/well and cultured as described above. Following a 24-hour incubation, the medium was exchanged with OMEM containing 1 μM DB compound or 0.1% DMSO (vehicle control). As previously described (24), cells were collected from each well after a 96-hour treatment and plated at an estimated density of 20,000 cells/well into a 96-well enzyme-linked immunosorbent spot (ELISpot) plate (MultiScreen-IP 0.45-μm filter plate; Millipore); prior to plating cells, each ELISpot well membrane was activated by a 2-min incubation with 70% ethanol. Plated cells were vacuum suctioned onto the membrane, after which plates were dried for 1 h at 50°C and then stored upside down at 4°C.

PrP^Sc^-positive cells were later identified by the SSCA, performed as previously described (44), except that cells were not treated with phenylmethylsulfonyl fluoride (PMSF) following proteinase K (PK) digestion. Additional modifications included the use of 0.2% nonfat dry milk (Bio-Rad) in 1× Tris-buffered saline (TBS) for blocking, 0.5 μg/ml of the anti-PrP monoclonal antibody 6H4 (Prionics) in 1× TBS plus 0.1% Tween 20 (TBST) and 0.5% nonfat milk powder for detection of PrP^Sc^, and an alkaline-phosphatase-conjugated anti-mouse IgG1 antibody (Southern Biotechnology Associates) diluted 1:2,000 in 1× TBST and 0.5% nonfat milk powder for the secondary antibody. PrP^Sc^-positive cells were detected as spots and counted by an ELISpot plate reader (AID ELISpot software version 5.0; AutoImmun Diagnostika, Germany).

The reduction in PrP^Sc^ was expressed as a percentage of the spot number in wells containing DB compound-treated cells relative to that in vehicle control-treated cells. The average percent reduction following DB compound treatment was determined from at least three independent experiments and statistically compared using a one-way ANOVA and Dunnett's multiple-comparison test for *post hoc* comparison of each compound to the vehicle control or the cutoff value of 68% reduction (*P*_Dun_ < 0.05; SigmaPlot ver. 13). The effects of each compound on PrP^Sc^ accumulation at 1 μM were further compared for the structure-activity relationship analysis using a one-way ANOVA and the Bonferroni method for *post hoc* pairwise multiple comparisons (*P*_Bon_ < 0.05; SigmaPlot ver. 13).

The SSCA was used to estimate the DB compound concentration that reduces PrP^Sc^ accumulation by 50% (i.e., the 50% tissue culture-effective concentration [TC-EC_50_]). This was performed using a 1/4-log dilution series ranging from 1 μM to 0.1 μM (DMSO working concentration was 0.1%). The TC-EC_50_ was estimated from the general logistic function fit to concentration-response data by nonlinear regression (SigmaPlot ver. 13). The mean TC-EC_50_ was determined from a minimum of three independent trials and statistically compared using a one-way ANOVA and the Bonferroni method for *post hoc* pairwise multiple comparisons (*P*_Bon_ < 0.05; SigmaPlot ver. 13).

### Cell proliferation.

To complement the WST-1-based cytotoxicity assays, the bromodeoxyuridine (BrdU) cell proliferation assay was used to measure the effect of preliminary hit DB compounds (1 μM) on cell proliferation throughout the period of treatment. PrP^Sc^-positive cells were passaged, plated, and treated as previously described for the WST-1 assay, except that mitotic activity was measured on each day of the 4-day treatment using a colorimetric BrdU cell proliferation enzyme-linked immunosorbent assay (ELISA) kit (Abcam, Cambridge, MA). Briefly, BrdU was added to wells 24 h prior to measurement. Cells were then fixed, and the plates were stored at 4°C until measurement. Incorporation of BrdU (i.e., cell proliferation) was quantified according to the manufacturer's instructions by measuring the ODs at 450 and 550 nm using a microplate reader (SpectraMax 190; Molecular Devices). The absorbance values (OD_450-550_) from a total of three independent experiments were mean centered and autoscaled as previously described (45). Cell proliferation was expressed as the average percent BrdU incorporation in DB compound-treated cells relative to that in the day-matched, vehicle control-treated (0.1% DMSO) cells. Statistical comparison was made using a one-way ANOVA and Dunnett's multiple-comparison test for *post hoc* comparison of day-matched DB compound treatments with the vehicle control (*P*_Dun_ < 0.05; SigmaPlot ver. 13).

In an additional experiment, the total number of cells was directly counted at the end of the 4-day treatment period. Briefly, cells were plated into the wells of an ELISpot plate and dried as previously described for the SSCA. Cells were subsequently stained with trypan blue and counted with an ELISpot plate reader (24). The percentages of cell counts from DB-compound treated wells relative to vehicle control-treated wells were statistically compared as described above.

### *PRNP* transcript levels and total PrP^C^.

Preliminary hit compounds at 1 μM were evaluated for effects on *PRNP* transcript levels using quantitative reverse transcription-PCR (qRT-PCR). PrP^Sc^-negative cells were treated and collected as described above and washed with 1× Dulbecco's PBS (D-PBS). Cell pellets were frozen at −80°C until further testing, upon which pellets were lysed in RTL buffer (Qiagen, Valencia, CA). Lysates were subsequently shredded using QIAshredder spin columns, and total RNA was collected using the RNeasy minikit according to the manufacturer's instructions (Qiagen, Valencia, CA). Total RNA was quantified by spectrophotometry (Thermo Scientific; ND-1000 spectrophotometer), and 10 μg of each sample was treated with DNase using the DNA-free DNA removal kit (Ambion, Austin, TX). Reverse transcription of 1 μg of RNA into cDNA was performed using the SuperScript III first-strand synthesis Supermix for qRT-PCR (Invitrogen, Carlsbad, CA). *PRNP* transcripts were quantified by real-time PCR using the CFX96 detection system (Bio-Rad) in 20-μl reaction mixtures containing 10 μl of SsoAdvanced Universal SYBR Green Supermix (Bio-Rad), 0.2 μM (each) primer specific for *PRNP* (24), and 2 μl of cDNA diluted 1:100 into water. Run conditions consisted of 95°C for 30 s, 35 cycles of 95°C for 5 s, and 60°C for 10 s, followed by a melt curve at 0.5°C increments from 60°C to 95°C. A standard curve generated from untreated PrP^Sc^-negative cells was used to transform threshold cycle (*C_T_*) values to relative concentrations of *PRNP. PRNP* transcripts were normalized to those of *GAPDH*, and fold change values in *PRNP* from compound-treated cells compared to the vehicle control were calculated by REST 2009 (V2.0.13; Qiagen). The fold changes in *PRNP* were mean centered and autoscaled as previously described (45), and the average fold change for each sample, from at least three independent experiments, was statistically compared to the vehicle control using a two-tailed *t* test (*P* < 0.05; SigmaPlot ver. 13).

Compounds at 1 μM were further evaluated for effects on PrP^C^ content using a commercial ELISA (TeSeE SAP detection kit; Bio-Rad) according to the manufacturer's instructions, but for quantification of PrP^C^, the PK digestion step was omitted, as previously described (24). PrP^Sc^-negative cells were collected by lifting with 500 μM EDTA in 1× PBS and centrifugation at 1,000 × *g* (JA50.5 rotor) for 7 min at 4°C. Cell pellets were lysed in lysis buffer supplemented with a protease inhibitor cocktail tablet (Roche Applied Science) for 3 min with gentle rocking on ice. Following centrifugation at 2,300 × *g* for 5 min at room temperature, lysates were stored at −20°C. Lysates were measured for total protein by the bicinchoninic acid protein assay kit (Thermo Scientific) and were appropriately diluted to allow equivalent protein concentrations within each sample added to the ELISA plate. A PrP^C^ standard curve was prepared using 1/2-log dilutions of ovinized RK13 (Rov) cell (46) lysates to transform corrected optical densities into a relative concentration of PrP^C^. Levels of total PrP from compound-treated cells were normalized to vehicle-treated cells. The change in total PrP was mean centered and autoscaled, and values were statistically compared as described above. Twofold changes in *PRNP* transcript levels or total PrP were considered biologically significant.

### PrP^Sc^-seeded misfolding assay.

Compounds were evaluated for effects on PrP^Sc^-seeded misfolding using a serial protein misfolding cyclic amplification (sPMCA) assay based on methods previously described (47). Briefly, a 10% mouse normal brain homogenate (mNBH) was prepared from tg338 mice which express ovine valine-136 haplotype PrP^C^ and served as the PMCA substrate. A 10% scrapie brain homogenate in conversion buffer (ScBH; genotype 136VV), the PMCA seed, was prepared from the hindbrain of a sheep displaying clinical signs of scrapie at cull and confirmed positive by Western blotting for PrP^Sc^ based on methods described below. The 10% ScBH was serially diluted into mNBH to provide PMCA seed dilutions of 10^−4^, 10^−5^, and 10^−6^. Ten microliters of each dilution was added to 0.2-ml reaction tubes and treated with the indicated concentration of each compound diluted into sterile double-distilled water (ddH_2_O). mNBH was added to achieve a final reaction volume of 100 μl. DMSO at a final concentration of 0.4% in mNBH served as the untreated vehicle control. The treated reaction mixtures were inserted into a microplate horn sonicator (Misonix S-4000; Qsonica) and maintained at 37°C in 30% Koolance by a compact recirculating chiller (Qsonica). After an initial 30-min incubation, reaction mixtures underwent PMCA cycling that consisted of 24-hour rounds of 48 cycles (30-s sonication [180 to 200 W] followed by 29.5-min incubation). Between each round, samples were diluted 1:3 into fresh mNBH containing the appropriate DB compound at the final concentration. A total of four rounds of PMCA were performed, and all reaction mixtures were stored at −80°C until tested for PK-resistant PrP^Sc^ by Western blotting.

Western blot detection of PMCA-generated PrP^Sc^ was performed as previously described (47, 48). Briefly, PMCA samples were incubated with 200 μg/ml (2.5 units/mg; Roche Applied Science) of PK at 37°C for 90 min. Following electrophoresis, PK-resistant PrP^Sc^ bands were detected using 3.5 μg/ml of the monoclonal antibody F99/97.6.1 (VMRD, Inc.) and a goat anti-mouse IgG1 antibody conjugated to horseradish peroxidase (HRP; 1:5,000; Southern Biotechnology Associates). The membrane was incubated with chemiluminescent substrate (Luminata Forte Western HRP substrate; Millipore) for 1 min, and signals were captured on autoradiography film (GeneMate) or digitally acquired using a Gel Doc imaging system (Molecular Imager ChemiDoc XRS system; Bio-Rad). Images were obtained using Quantity One software (Bio-Rad), and PK-resistant PrP^Sc^ band intensities were measured by densitometry using the image processing software Fiji (version 1.50a; http://fiji.sc/) (49 – 51). PMCA rounds were normalized to the initial round of PrP^Sc^ detection in the vehicle control (i.e., the initial round of detection was designated round 1_n_). Treated reaction mixtures were compared to the vehicle control to calculate the percent reduction in PrP^Sc^. A percent reduction categorical score was given based off the percent reduction in band intensity at a given concentration, seed, and round. For each score, 0 represented no inhibition (i.e., percent reduction of <43%), 1 represented at least a 1/4-log reduction in seeded misfolding (i.e., percent reduction between 44% and 67%), and 2 represented at least a 1/2-log reduction in seeded misfolding (i.e., percent reduction exceeds the established cutoff of ≥68%).

### PrP^Sc^ stabilization against PK digestion.

Compounds were evaluated for effects on PrP^Sc^ stabilization against digestion with PK. Hindbrain tissues collected from a scrapie-positive sheep were homogenized in lysis buffer (0.5% NP-40, 0.5% deoxycholic acid, 1× PBS) to a final concentration of 10% (wt/vol). Homogenates were assessed for total protein by the bicinchoninic acid protein assay kit (Thermo Scientific) and were appropriately diluted to a final concentration of 2 mg/ml in 0.32 M sucrose in 1× PBS. Stock compounds were prepared by diluting to the appropriate concentration (range, 953 to 29.8 μM) into 100% DMSO. Stocks were aliquoted and stored at −20°C. Two microliters of stock compounds was diluted to the final working concentration (range, 38.1 to 1.2 μM) into 48 μl of brain homogenate for a final sample volume of 50 μl. DMSO at a final concentration of 4% in brain homogenate served as the untreated vehicle control. Samples were incubated at 37°C for 30 min in a Thermomixer R (Eppendorf North America, Inc.) set at 700 rpm. Following incubation, PK was added to each sample to a final concentration of 50 μg/ml and samples were incubated at 37°C, with shaking at 700 rpm, for 1 h. Following PK digestion, 4× lithium dodecyl sulfate loading buffer (NuPAGE Novex; Thermo Fisher Scientific) was added, and the samples were boiled for 5 min. Gel electrophoresis and membrane transfer were performed as previously described (52), and immunoblotting and image analysis were performed as described above. PK-resistant PrP^Sc^ band intensities from compound-treated samples were normalized to that of the vehicle control to calculate the relative percent PrP^Sc^, and the relative percent PrP^Sc^ of each sample was mean centered as previously described (45). The average relative percent PrP^Sc^, from at least 5 independent experiments, was statistically compared to the vehicle control using a two-tailed *t* test (*P* < 0.05; SigmaPlot ver. 13).

## RESULTS AND DISCUSSION

### Effects on ovine microglia viability.

**FIG 1:**
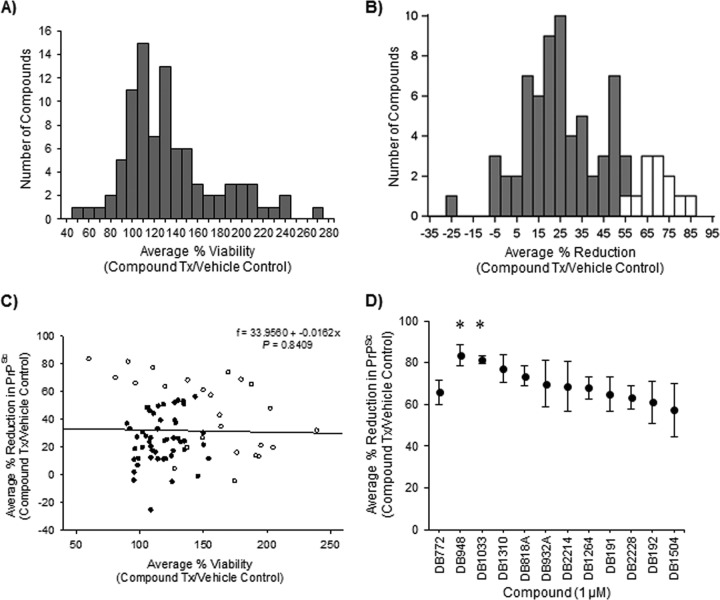
Identification of DB compounds inhibitory at 1 μM in PrP^Sc^-positive hTERT-ovine microglia. Following 4 days of incubation with 1 μM (each) DB compound, cell viability and PrP^Sc^ inhibition were evaluated by WST-1 assay and SSCA, respectively. Values were expressed as the percent change in viability or PrP^Sc^ spot number versus the vehicle control (0.1% DMSO). (A) Distribution of tested compounds with regard to effects on cell viability. Values along the *x* axis represent the mean percent viability from a minimum of three independent experiments. Values along the *y* axis represent a number of the total compound population that falls within a value of percent change. Compounds were compared to the vehicle control, and those lacking significant effects on cell viability or of distinct structural interest were selected for inhibition screening. (B) Distribution of selected compounds with regard to effects on PrP^Sc^ accumulation. Values along the *x* axis represent the mean percent change in spot number from a minimum of three independent experiments. Values along the *y* axis represent the number of the total compound population that falls within a value of percent change. Compounds with a percent reduction of greater than or equal to 68% (+ standard deviation) were identified as preliminary hits and selected for further testing (white bars). (C) Correlation between average percent reduction in PrP^Sc^ and average percent viability following 1 μM treatment with DB compounds with (open circles, *P*_Dun_ < 0.05) or without (closed circles) significant effects on cell viability. The black line represents the best fit line following linear regression analysis. (D) Average percent reduction in PrP^Sc^ by the 12 preliminary hits selected from panel B. Values were statistically compared to that of the reference molecule, DB772, to determine enhanced inhibitory activity (*, *P*_Dun_ < 0.05). Error bars represent ±1 standard deviation of the mean from at least 3 independent experiments.

When screening candidate antiprion compounds, it is critical to recognize those that significantly alter cell viability, as such an effect may influence the measurement of PrP^Sc^ accumulation. For example, increasing the rate of cell division decreases the rate of PrP^Sc^ accumulation in murine neuroblastoma cells (53). Conversely, cytotoxic effects would reduce the number of cell targets and may decrease PrP^Sc^ replication and accumulation. Thus, DB compounds were initially screened for effects on the viability of hTERT-ovine microglia under the same culture conditions subsequently used to screen for antiprion activity (i.e., viability was measured by WST-1 assay 4 days post-exposure to 1 μM DB compound). Consistent with our previous findings (24), 1 μM DB772 did not significantly affect cell viability (96.4% ± 8.7% of vehicle control). Cell viability was significantly decreased by one DB compound (DB948; 60%) and increased by 24 DB compounds (range, 127 to 266%); however, the majority (64 total) demonstrated no significant effect when tested at 1 μM (Fig. 1A). Of the DB compounds selected for further testing, 59 had no significant micromolar effect on cell viability (range, 76 to 156%). Seventeen additional DB compounds had significant effects on cell viability but were also selected for further testing because of distinct structural interests: one significant inhibitor (60%) and 16 significant enhancers (range, 127 to 239%).

### Effects on persistent scrapie infection of ovine microglia.

The selected DB compounds were next screened for effects on persistent scrapie infection of hTERT-ovine microglia as determined by SSCA. After 4 days of incubation, 1 μM DB772 significantly reduced PrP^Sc^-positive spots by 65.8% ± 5.7% (*P*_Dun_ < 0.001). This activity was consistent with our previous finding in which DB772 at 4 μM resulted in a nearly complete inhibition of PrP^Sc^ accumulation in scrapie-infected primary ovine microglia (24). The results for the 76 selected DB compounds are summarized in Fig. 1B as the distribution of the mean relative reductions in PrP^Sc^-positive microglia. In addition to DB772, 24 other DB compounds significantly reduced PrP^Sc^-positive spots (range, 43 to 83%; *P*_Dun_ < 0.05). Six of these were also shown to affect cell viability under these same culture conditions, one of which decreased (60% of vehicle control) and 5 of which increased (range, 150 to 203%) cell viability. However, the magnitudes of the antiprion and cell viability effects were not correlated (slope of the linear regression = −0.0162 ± 0.0803, *P* = 0.8409) (Fig. 1C), suggesting that the antiprion effects of these DB compounds were not simply caused by an associated effect on cell viability. Compounds that reduced PrP^Sc^ to a percentage of the vehicle control that was ≥68% were selected as preliminary hit compounds. The antiprion effects of 12 DB compounds identified as preliminary hits are shown in Fig. 1D. At the working concentration of 1 μM, only DB948 (83%) and DB1033 (81%) produced significantly greater antiprion activity than DB772.

### Effects on ovine microglial proliferation.

**FIG 2:**
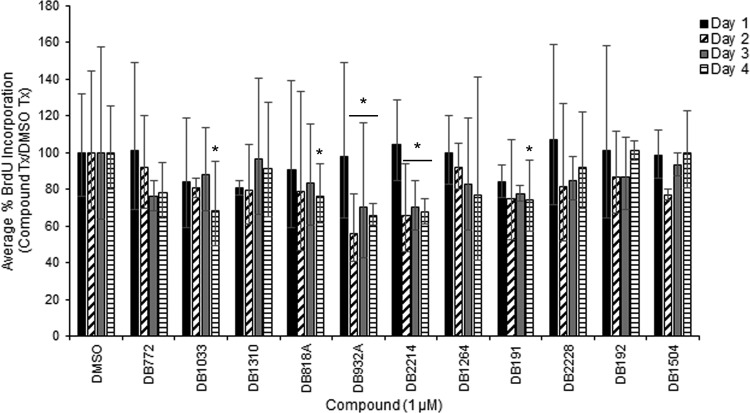
Effect of preliminary hit DB compounds on cell proliferation. PrP^Sc^-positive hTERT-ovine microglia were treated for 4 days with 1 μM DB compound, during which cell proliferation was quantified daily by 24-h BrdU incorporation. Bars represent the average percent BrdU incorporation in DB compound-treated cells relative to that in day-matched, vehicle control (0.1% DMSO)-treated cells. Error bars represent the 95% confidence intervals from the averages from 3 independent experiments. Values were statistically compared to the day-matched vehicle controls to determine enhanced or reduced cell proliferation (*, *P*_Dun_ < 0.05).

The effects of DB compounds on antiprion activity were not solely attributed to cell viability; however, it remained possible that additional effects on cell proliferation had impacted PrP^Sc^ accumulation (53). Therefore, the preliminary hit DB compounds were examined for effects at 1 μM on the proliferation of hTERT-ovine microglia throughout the 4-day course of treatment. Given its large cytotoxic effect, the effect of 1 μM DB948 on cell proliferation was not tested. A significant effect on cell proliferation was not detected over the 4-day treatment period for DB772, DB1310, DB1264, DB2228, DB192, and DB1504 (range, 76 to 106% of vehicle control) (Fig. 2). Conversely, significant decreases in cell proliferation were detected on days 2 through 4 following treatment with DB932A (range, 56 to 70%; *P*_Dun_ < 0.05) and DB2214 (range, 66 to 70%; *P*_Dun_ < 0.01). Significant decreases were detected only on day 4 following treatment with DB1033 (68%; *P*_Dun_ = 0.003), DB818A (76%; *P*_Dun_ = 0.048), and DB191 (74%; *P*_Dun_ = 0.027). Thus, we cannot exclude the possibility that a reduced rate of cell division might contribute to the antiprion activity of some of these compounds. However, significant correlations were not detected between effects on cell proliferation and cell viability (*P* = 0.9893), cell number (*P* = 0.1930), and antiprion activity (*P* = 0.0731) after 4 days of treatment. This further suggests that while a subset of DB compounds may target cell division or viability, these effects are likely not the sole cause of PrP^Sc^ inhibition. A summary of the cell-based effects (i.e., PrP^Sc^ inhibition, cell counts, cell viability, and BrdU incorporation) of the preliminary hit DB compounds is provided in Table S2 in the supplemental material and highlights that compounds such as DB1310, which significantly inhibits PrP^Sc^ but has no detectable effect on cell count, viability, or BrdU incorporation, are compounds of future interest.

### Calculation of the selectivity index for preliminary hit compounds.

To compare levels of inhibition and cytotoxicity for the preliminary hit compounds, PrP^Sc^-positive hTERT-ovine microglia were exposed to compounds in a 1/4-log dilution series for 4 days and evaluated for cell viability and persistence of scrapie infection by the WST-1 assay and SSCA, respectively. The concentration inducing 50% cellular cytotoxicity (CC_50_) and the concentration inducing 50% inhibition of PrP^Sc^ (TC-EC_50_) were calculated.

**FIG 3:**
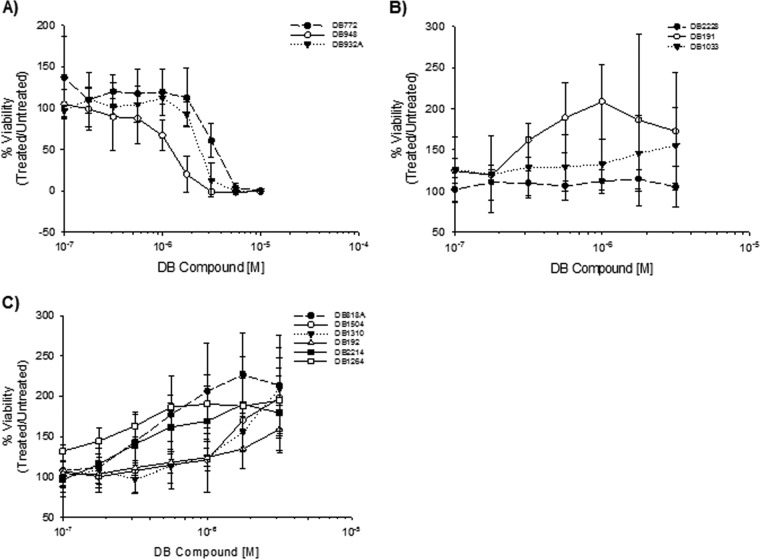
Dose-response curves show variable effect on cell viability by preliminary hit DB compounds. PrP^Sc^-positive hTERT-ovine microglia were treated for 4 days with a 1/4-log dilution series of each compound. Cell viability was subsequently determined by WST-1 assay. (A) DB compounds that exhibited a concentration-dependent cytotoxic effect. (B) DB compounds that did not induce a significant effect on cell viability up to a concentration of 3.16 μM. (C) DB compounds that led to a concentration-dependent increase in cell viability. Data points represent the mean percent viability ±1 standard deviation from at least 3 independent experiments.

Of the 12 DB compounds identified as preliminary hits, concentration-dependent cytotoxicity was observed for only three (Fig. 3A). The cytotoxicity of DB772 at a concentration greater than 1 μM is consistent with our previous finding, which used primary ovine microglia (24). The structures of DB948 and DB932A represent minimal chemical modifications of DB772; thus, it is not surprising that the CC_50_ values associated with these compounds were similar (Table 1). Significant cytotoxicity was not observed for the other 9 preliminary hit compounds, three of which (DB2228, DB191, and DB1033) failed to demonstrate any effect on cell viability (Fig. 3B) and six of which were associated with a concentration-dependent increase in cell viability (Fig. 3C). The CC_50_ for these 9 preliminary hit compounds was thus nonestimable and is instead reported as >3.16 μM—the highest concentration tested.

**TABLE 1:**
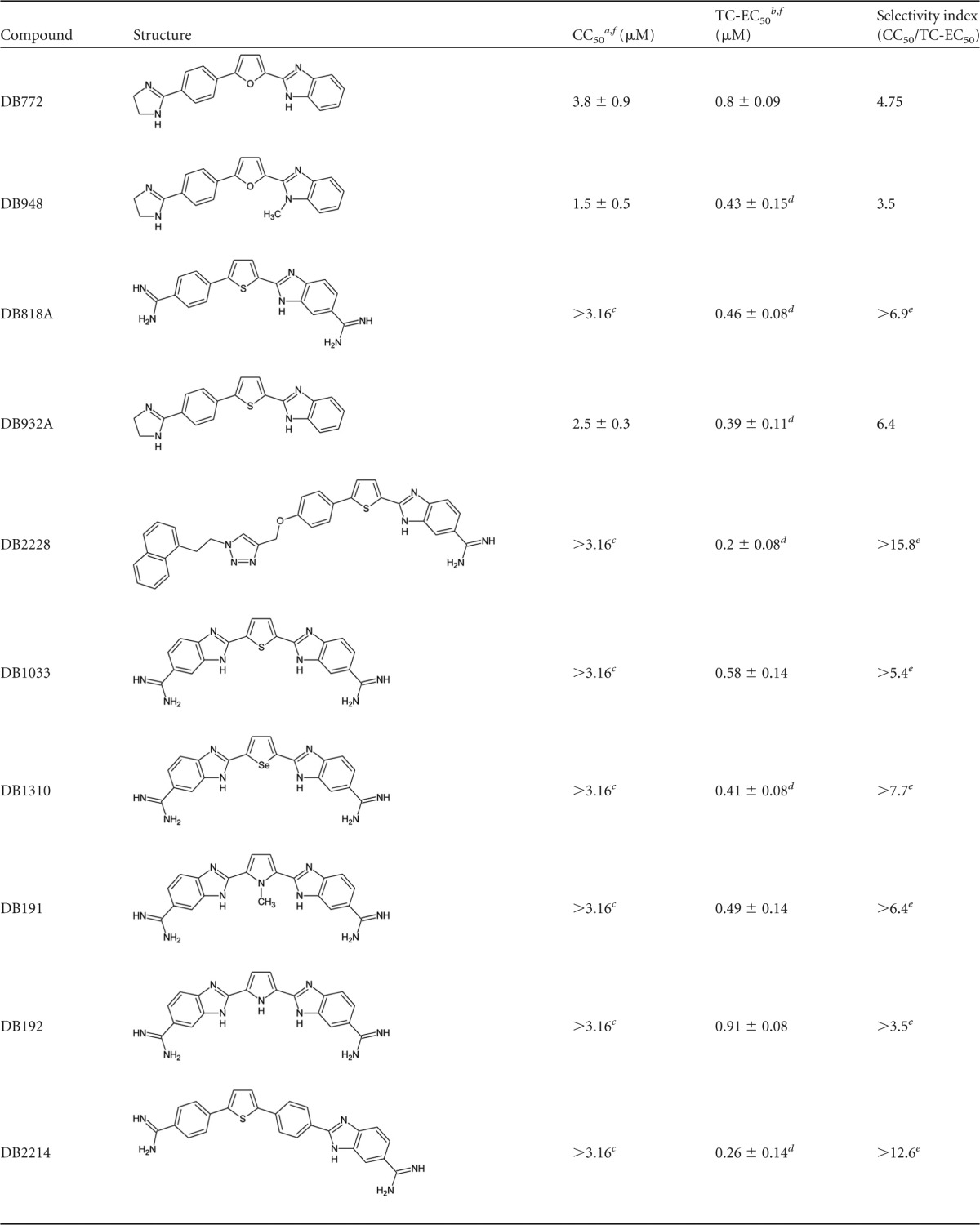

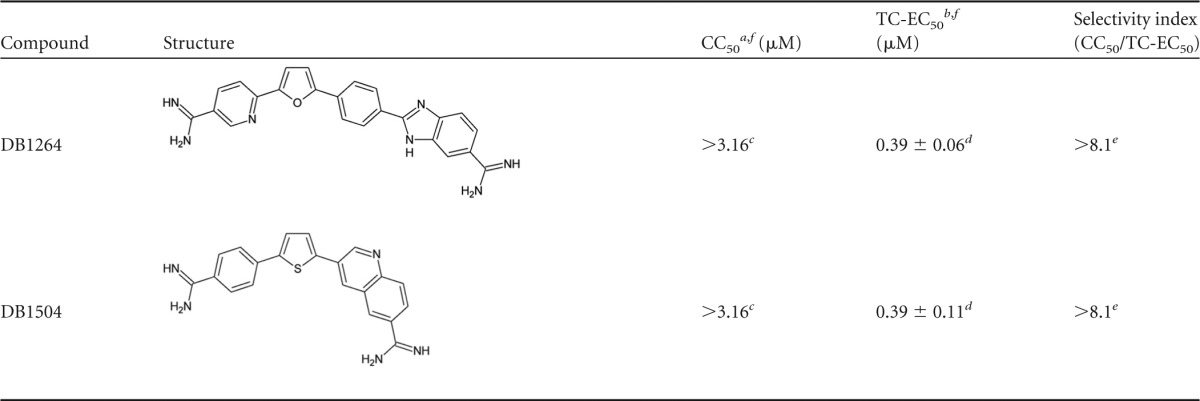
Comparison of antiprion activities and cytotoxicities of preliminary hit compounds

a CC_50_, 50% cytotoxic concentration.

b TC-EC_50_, 50% tissue culture effective concentration.

c >3.16, cytotoxicity curves did not reach 50% inhibition at the highest concentration tested.

d TC-EC_50_ is statistically lower than that for DB772 (*P*_Bon_ < 0.05).

e >, selectivity index is greater than the indicated value.

f Values are the mean ± 1 standard deviation from at least 3 independent experiments.

Concentration-dependent antiprion activity was observed for all 12 preliminary hit compounds. For each of the compounds, the estimated TC-EC_50_s (Table 1) were derived from the best-fit sigmoidal response curves. As an initial measure relating potential therapeutic safety to antiprion efficacy, the tissue culture selectivity index (SI) was calculated (CC_50_/TC-EC_50_) (Table 1). Consistent with our previous finding using primary ovine microglia (24), the TC-EC_50_ of DB772 using hTERT-ovine microglia was 0.8 ± 0.09 μM, resulting in a selectivity index of 4.75. Although the TC-EC_50_s for DB948 and DB932A were significantly lower than that estimated for DB772, these structural modifications conferred no (DB948 = 3.5) or only modest (DB932A = 6.4) improvement in the selectivity index. Of the remaining preliminary hit compounds, the TC-EC_50_s of six were significantly lower than that estimated for DB772 and three were statistically equivalent. We cannot rule out the possibility that enhanced cell viability may have contributed in part to the more potent antiprion activity of some of these preliminary hit compounds (e.g., DB191), but enhanced viability was unlikely to have contributed significantly to the antiprion activities of DB1504 and DB1310 since the TC-EC_50_s for these compounds (0.39 ± 0.09 μM and 0.41 ± 0.08 μM, respectively) were at least a half-log lower than the lowest concentrations causing enhanced cell viability (between 1 and 1.78 μM for both). Of particular interest is the potent antiprion activity of DB2228, which had the lowest TC-EC_50_ (0.2 ± 0.08 μM) of all preliminary hit compounds and no effect on cell viability up to a concentration of 3.16 μM.

### Effects of antiprion compounds on *PRNP* transcript levels and total PrP^C^.

**FIG 4:**
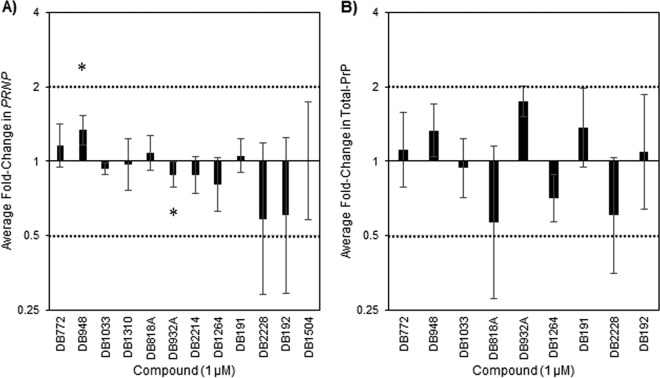
Prion protein expression is not inhibited by preliminary hit DB compounds. The effects of preliminary hit DB compounds on *PRNP* transcript levels (A) and total PrP (B) were evaluated. (A) RNA was collected from treated cells and tested by qRT-PCR for *PRNP* transcript levels. *PRNP* transcripts were normalized to *GAPDH* transcript and compared to the vehicle control. (B) Total protein was collected from treated cells and tested for total PrP^C^ using a commercial ELISA. Total PrP was normalized to the vehicle control. Columns represent the fold change in *PRNP* transcripts or total PrP. Increasing bars indicate compound enhancement, and decreasing bars indicate compound inhibition. Fold change values were statistically compared to the vehicle control using individual *t* tests (*, *P* < 0.05). The *y* axis reference lines indicate the established 2-fold cutoff level for biologically significant inhibition or enhancement. Error bars represent the 95% confidence intervals from the mean fold change from at least 3 independent experiments.

To elucidate a possible mechanism of inhibition, the preliminary hit compounds were examined for effects on PrP^C^ expression. Evidence has supported the reliance of prion replication on multiple PrP^C^-specific factors, including *PRNP* expression and total PrP^C^ (46, 54). Therefore, a reduction in PrP^C^ expression represents a possible target for antiprion compounds. To determine if PrP^C^ expression is inhibited, the effects on total cellular content of PrP transcripts (qRT-PCR) (Fig. 4A) and protein (ELISA) (Fig. 4B) were determined in PrP^Sc^-negative cells after 4 days of incubation with preliminary hit compounds at 1 μM. Results were normalized to vehicle-treated cells, and fold changes were statistically compared to the vehicle control. Consistent with our previous finding (24), a significant effect on PrP^C^ expression was not detected for DB772. None of the remaining 11 preliminary hit compounds caused a 2-fold change in either *PRNP* transcript or total PrP levels. The only statistically significant changes were in *PRNP* transcripts after incubation with DB948 (*P* = 0.0262) and DB932A (*P* = 0.0467), although both were less than 2-fold. Significant correlations of antiprion activity at 1 μM with *PRNP* transcript (*P* = 0.1084) or total PrP^C^ (*P* = 0.8333) levels were not detected, strongly suggesting that inhibition of PrP^C^ expression is not the general mechanism by which these selected DB compounds inhibit PrP^Sc^.

### Effects of preliminary hit compounds on PrP^Sc^-seeded misfolding.

Another potential mechanism of antiprion activity is through interference with the proposed mechanism of prion replication, that is, interference with PrP^Sc^-seeded misfolding of PrP^C^ to nascent PrP^Sc^. To determine the effect of DB772 directly on PrP^Sc^-seeded misfolding, serial protein misfolding cyclic amplification (sPMCA) assays were performed. Reaction mixtures were treated with DB772 prior to each round using concentrations that previously demonstrated significant anti-PrP^Sc^ activity in cell culture (1 and 4 μM). Serial PMCA was carried out for four rounds, and reaction mixtures were subsequently tested for PK-resistant PrP^Sc^ by Western blotting. To account for variation between assays, samples were normalized to the initial round of PrP^Sc^ detection in the vehicle control: for each assay, the initial round of detection was designated round 1_n_. Activity for each concentration at a given seed dilution and round was scored based on the percent reduction in band intensity.

**FIG 5:**
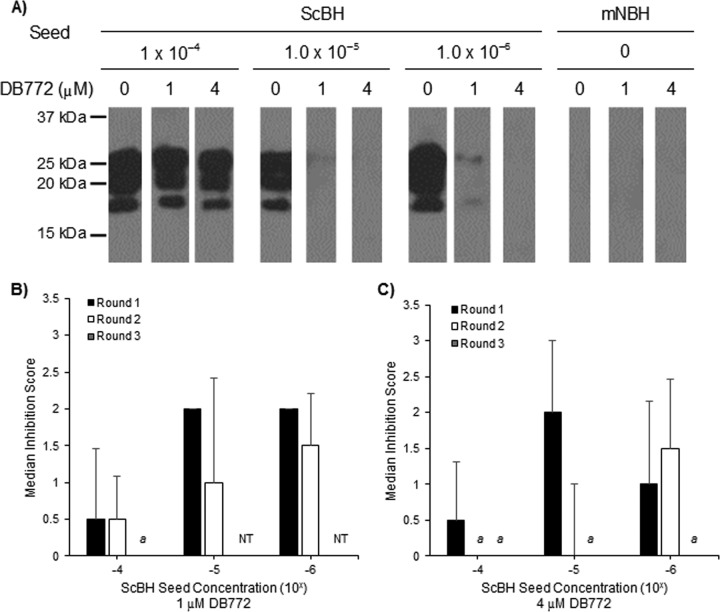
DB772 inhibits PrP^Sc^-seeded misfolding during serial PMCA assay. The inhibition of PrP^Sc^-seeded misfolding was evaluated by sPMCA assay by treating mNBH with either 1 or 4 μM DB772. Treated reaction mixtures were seeded with 10^−4^, 10^−5^, or 10^−6^ dilutions of 10% ScBH and processed for 4 rounds. Reaction mixtures were collected at each round and tested for PK-resistant PrP^Sc^ by immunoblotting. PMCA rounds were normalized to the initial round of PrP^Sc^ detection (i.e., the initial round of detection corresponds to round 1_n_). (A) A representative immunoblot from samples collected at round 1_n_ is demonstrated. Band intensities were quantified by densitometry. (B and C) Densities of bands from DB772-treated reaction mixtures were normalized to those of vehicle control (0.4% DMSO) to obtain a percent reduction categorical score for DB772 at 1 μM (B) and 4 μM (C). Data columns represent the median score ±1 standard deviation. *a*, all three replicates scored 0. NT, reaction mixtures seeded with 10^−5^ or 10^−6^ ScBH and treated with 1 μM DB772 were not tested at round 3_n_.

PrP^Sc^-seeded misfolding was inhibited in the presence of DB772 at both 1 and 4 μM (Fig. 5A). While the inhibition was most pronounced for reaction mixtures using more dilute PrP^Sc^ seed (i.e., in reaction mixtures using 10^−5^ and 10^−6^ dilutions of ScBH) (Fig. 5B and C), the inhibitory effect of 1 μM DB772 was similar to, if not greater than, that at 4 μM. This suggests that micromolar DB772 is near its maximal effective concentration in this assay, which is consistent with its micromolar maximum antiprion activity in cell culture. It is interesting that the initial inhibitory action of DB772 was overcome with additional rounds of PMCA despite the fresh addition of DB772 at the beginning of each round. This indicates that micromolar DB772 may inhibit only a subpopulation of the seeds present in ScBH, thereby delaying rather than abolishing the accumulation of PK-resistant PrP^Sc^ in this assay. It is also possible that DB772 may select for a quasispecies more resistant to this inhibitory activity, similar to what has been observed with some antiprion compounds (e.g., swainsonine) (55). Nevertheless, 1 μM DB772 did reduce PrP^Sc^-seeded misfolding by 1/2-log or greater (i.e., a score of 2) compared to the vehicle control, indicating that DB772 may directly inhibit PrP conversion.

**FIG 6:**
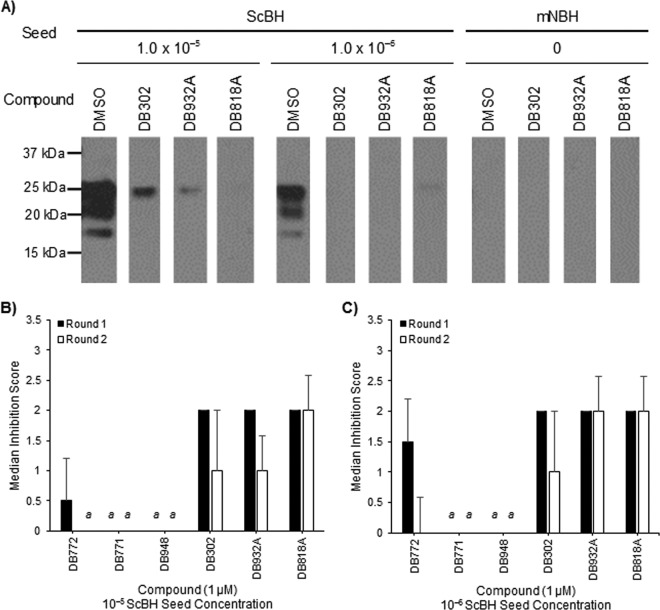
Selected DB compounds inhibit PrP^Sc^-seeded misfolding during serial PMCA assay. The inhibition of PrP^Sc^-seeded misfolding was evaluated by sPMCA assay by treating mNBH with 1 μM indicated compound. Reaction mixtures were collected at each round and tested for PK-resistant PrP^Sc^ by immunoblotting, and band intensities were analyzed as previously described (Fig. 5). (A) A representative immunoblot from samples collected at round 2_n_ is demonstrated. (B and C) Percent reduction categorical scores were evaluated from reaction mixtures seeded with 10^−5^ (B) or 10^−6^ (C) dilutions of 10% ScBH. Data columns represent the median score ±1 standard deviation. *a*, all three replicates scored 0.

In the next experiment (Fig. 6), reaction mixtures were treated with 1 μM three preliminary hit compounds (DB818A, DB932A, and DB948), two structurally similar compounds that had demonstrated weak inhibitory activity in cell culture (DB771 and DB302), and DB772 as a control. DB771, which has only one substitution compared to DB772 and is a weak cell culture inhibitor, lacked any measurable inhibitory activity against PrP^Sc^-seeded misfolding. Interestingly, DB948, the compound with the strongest cell-based inhibitory activity, also lacked detectable inhibition in the sPMCA reaction mixtures. DB932A, DB818A, and the remaining weak cell culture inhibitor, DB302, demonstrated strong inhibitory activity against PrP^Sc^-seeded misfolding (Fig. 6A). The peak inhibitory activity of DB772 was observed at round 1_n_, particularly in reaction mixtures seeded with 10^−6^ dilutions of ScBH. DB932A and DB302 exhibited strong inhibition following round 1_n_, regardless of the seed concentration. Inhibition declined slightly following round 2_n_ but remained in reaction mixtures seeded with a 10^−6^ dilution of ScBH. Finally, DB818A inhibited PrP^Sc^-seeded misfolding in all reaction mixtures regardless of the seed concentration or round (Fig. 6). Taken together, the data suggest that DB772 and some of the structurally similar analogs may act by reducing prion-associated conversion of PrP^C^ to PrP^Sc^. As demonstrated by DB948 and DB302, the cell-based inhibitory activity does not always follow that of seeded misfolding inhibition, which may reflect alternative mechanisms of PrP^Sc^ inhibition (e.g., for DB948) and alternative cellular distribution and/or metabolism (e.g., for DB302).

### Stabilization of PrP^Sc^ against PK digestion by preliminary hit compounds.

**FIG 7:**
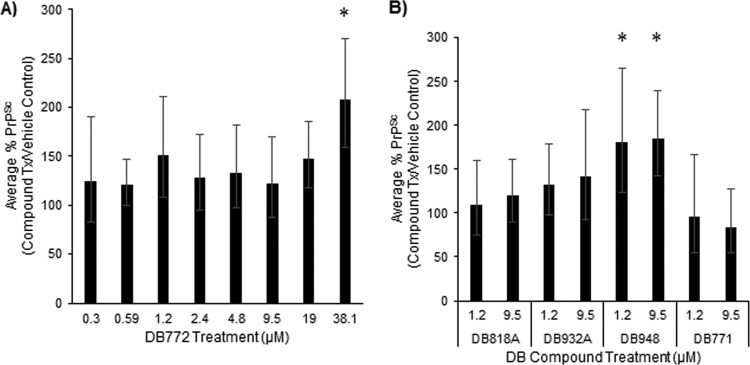
DB772 and DB948 stabilize PrP^Sc^ to reduce PK digestion. The stabilization of PrP^Sc^ and subsequent reduction in PK digestion by selected compounds were evaluated by treating scrapie-positive sheep brain homogenate with the indicated concentration and DB compound. Aliquots of brain homogenate were treated with the indicated dilutions of DB772 (A) or selected DB compounds (B) and tested for PK-resistant PrP^Sc^ by immunoblotting. Band intensities were quantified as described above and normalized to the vehicle control. Columns and error bars represent the mean relative percent PrP^Sc^ and 95% confidence intervals, respectively, from at least 5 independent experiments. Mean values were statistically compared to the vehicle control using individual *t* tests (*, *P* < 0.05).

As has been associated with LCPs (39), candidate antiprion compounds may also inhibit PrP^Sc^ accumulation through the stabilization of PrP^Sc^ amyloids. To determine the effect of preliminary hit compounds on the stability of PrP^Sc^ against PK digestion, a scrapie-positive sheep brain homogenate was treated with a dilution series of DB772 (range, 38.1 to 1.2 μM) similar to previous work done using polythiophenes (39). Relative to vehicle-treated controls, preincubation of ScBH with DB772 significantly increased PK-resistant PrP^Sc^ bands in samples treated with 38.1 μM (107% increase, *P* = 0.0018) (Fig. 7A) but not at the lower concentrations that were effective in inhibiting PrP^Sc^ accumulation in cell culture and sPMCA assays; it is possible that this disparity between effective concentrations may just reflect differences in assay matrices (e.g., abundance of lipids in brain homogenate, etc.), but this was not further investigated.

A similar experiment was conducted using three DB compounds more potent than DB772 (DB948, DB932A, and DB818A), the inactive compound DB771, and a vehicle control (4% DMSO). Similar to the biologically inactive DB771, significant stabilization of PrP^Sc^ was not detected for DB932A or DB818A at biologically relevant concentrations (Fig. 7B). Thus, the collective data regarding DB932A and DB818A suggest that a primary mechanism of potent antiprion activity is to reduce conversion of PrP^C^ to PrP^Sc^. In contrast, DB948, at both 9.5 μM (84% increase, *P* = 0.0145) and 1.2 μM (81% increase, *P* = 0.029), significantly increased the amount of PK-resistant PrP^Sc^ observed (Fig. 7B). The mechanism of DB compound-induced PrP^Sc^ stabilization appears to be a direct action on misfolded PrP rather than a generalized inhibition of PK since, similarly to the other DB compounds tested, pretreatment of ScBH with DB948 (see Fig. S2 in the supplemental material) or DB772 (data not shown) results only in discrete PK-resistant PrP^Sc^ bands with the expected shifts in apparent mass. Unlike DB772, DB948 lacked any effect on PrP^Sc^-seeded misfolding in the sPMCA assay, suggesting that induced stabilization of PrP^Sc^ can lead to reduced accumulation of PrP^Sc^ in cell culture by a mechanism independent of inhibiting seeded misfolding. Taken all together, we cannot be certain that a DB compound-induced stabilization of PrP^Sc^ is necessarily linked to inhibition of PrP^Sc^-seeded misfolding activity or to antiprion activity, and so we suspect that an additional mechanism may contribute to the antiprion activity of some of these compounds. For example, given its cytotoxicity, the antiprion activity of DB948 might also include inhibition of non-PrP targets or cofactors (e.g., phosphatidylethanolamine and glycosaminoglycan) that have been shown to play a role in prion pathogenesis (56, 57).

### Structure-activity relationship analysis.

Of the 89 DB library compounds initially studied, 22 were structurally related as phenyl-heterocyclic-benzimidazoles (Table 2), eight as bis-benzimidazoles (Table 3), and five as diarylthiophene or -furan compounds (Table 4). Of the 12 preliminary hit compounds (Table 1), five are phenyl-heterocyclic-benzimidazoles (DB772, DB948, DB818A, DB932A, and DB2228), four are bis-benzimidazoles (DB1033, DB1310, DB191, and DB192), and two are diarylthiophene or -furan compounds (DB2214 and DB1264). One additional preliminary hit compound was a structurally related phenyl-heterocyclic-quinoline (DB1504). The findings of this study suggest that two elements are important to the micromolar antiprion activity of the pharmacophore: a core element of multiple simply linked aromatic rings and at least one basic terminal group, such as an amidine or cyclic imidazoline.

**TABLE 2:**
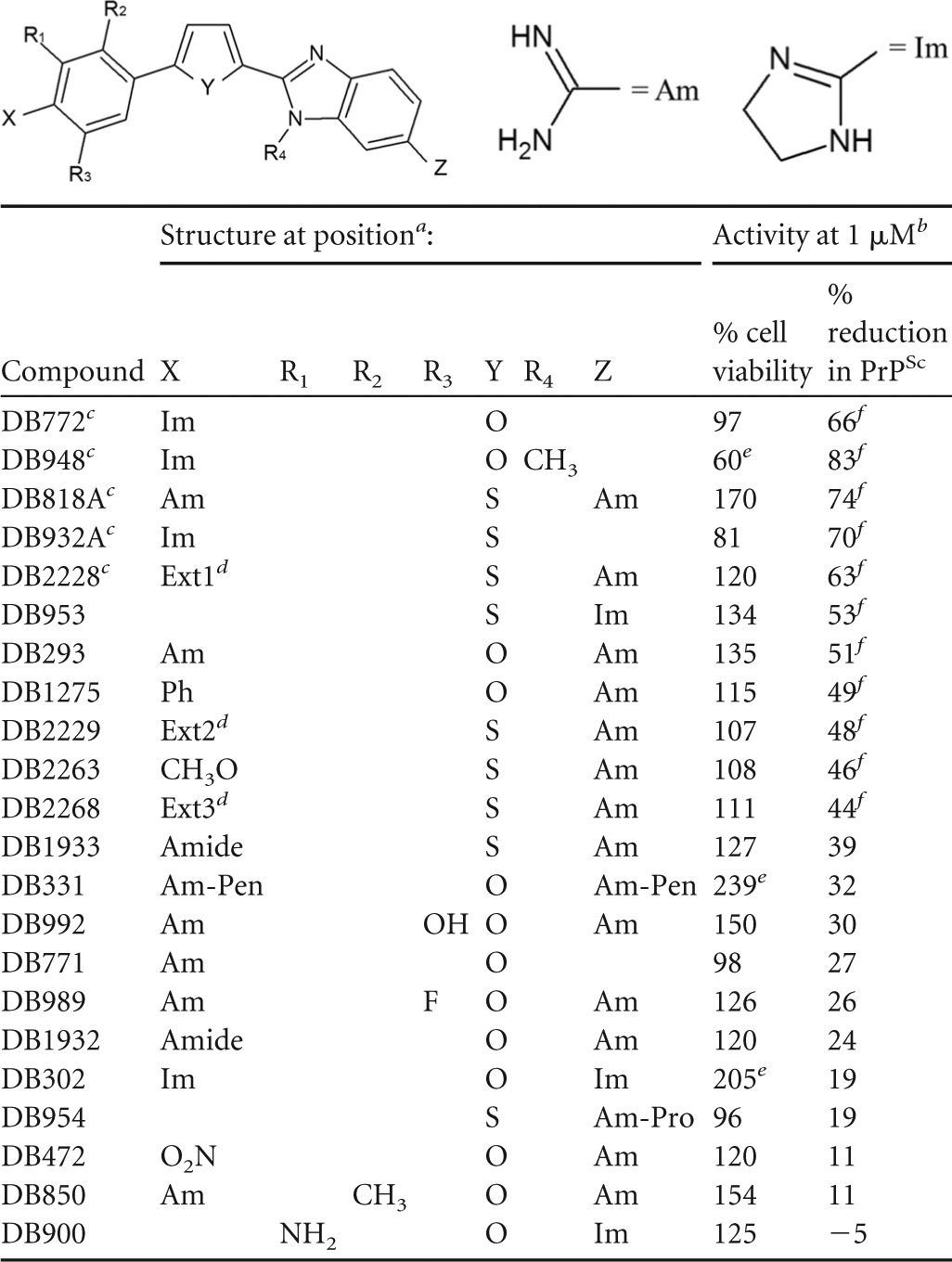
Structure and activities of phenyl-heterocyclic-benzimidazoles

a Ph, C_6_H_5_; Am-Pen, C(=NH)NH_2_-C_5_H_9_; Am-Pro, C(=NH)NH-C_3_H_7_.

b Average percent viability or reduction following 1 μM treatment from at least 3 independent experiments.

c Preliminary hit compound.

d For structures of Ext1, Ext2, and Ext3, see Table S3 in the supplemental material.

e Significant effect on cell viability (*P*_Dun_ < 0.05).

f Significant reduction in PrP^Sc^ accumulation (*P*_Dun_ < 0.05).

**TABLE 3:**
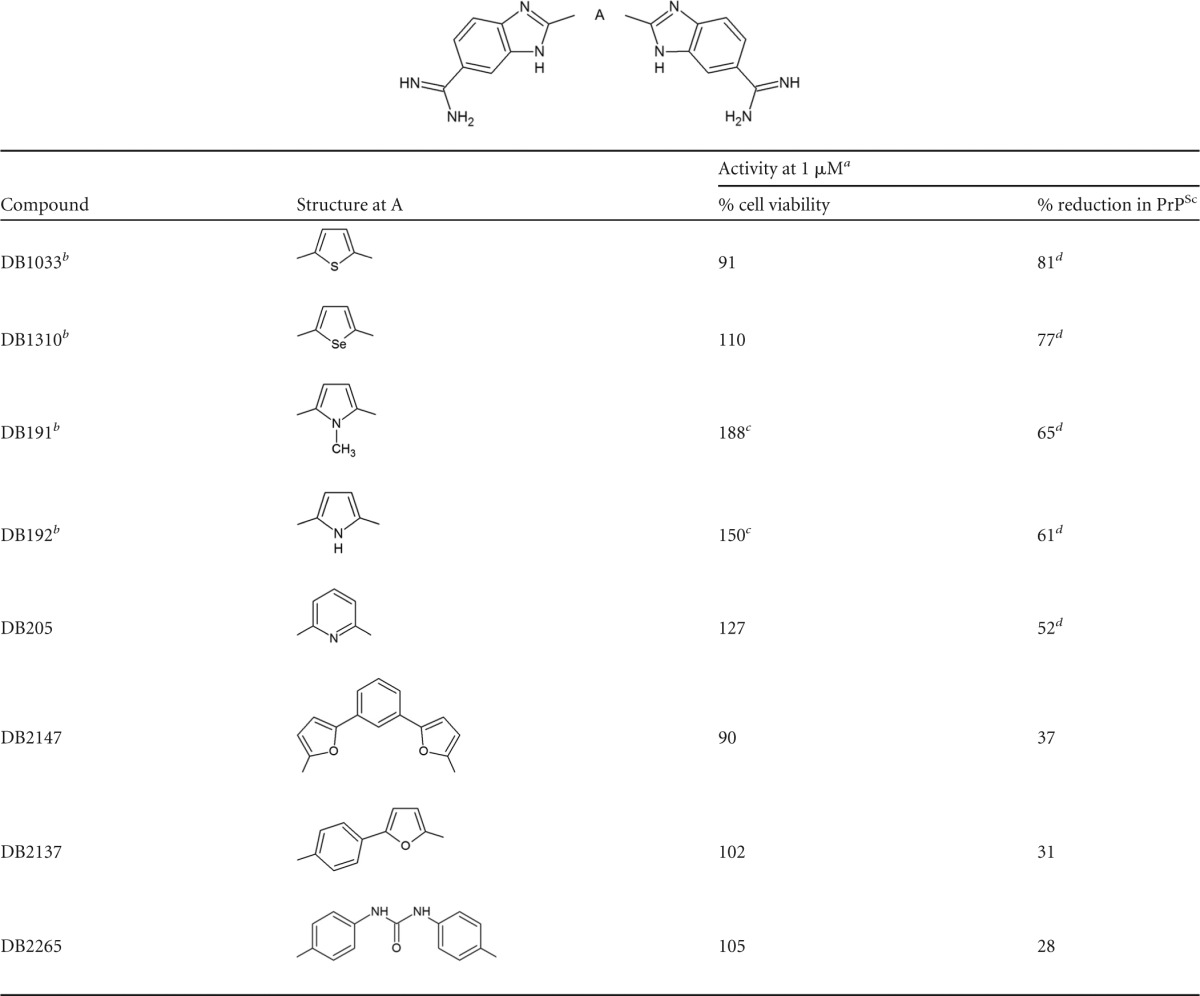
Structure and activities of bis-benzimidazoles

a Average percent viability or reduction following 1 μM treatment from at least 3 independent experiments.

b Preliminary hit compound.

c Significant effect on cell viability (*P*_Dun_ < 0.05).

d Significant reduction in PrP^Sc^ accumulation (*P*_Dun_ < 0.05).

**TABLE 4:**
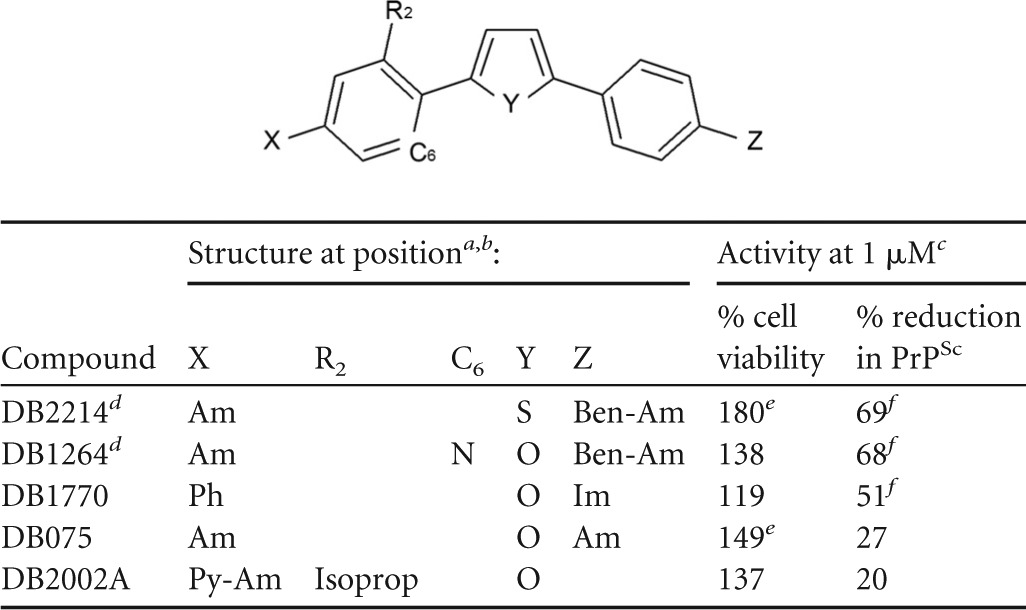
Structure and activities of diarylthiophene or -furan compounds

a For structures of Am and Im, see Table 2.

b Ben-Am, C_7_H_5_N_2_-(=NH)NH_2_; Ph, C_6_H_5_; Py-Am, C_5_H_4_N-(=NH)NH; Isoprop, C_3_H_7_O.

c Average percent viability or reduction following 1 μM treatment from at least 3 independent experiments.

d Preliminary hit compound.

e Significant effect on cell viability (*P*_Dun_ < 0.05).

f Significant reduction in PrP^Sc^ accumulation (*P*_Dun_ < 0.05).

DB772 and its thiophene equivalent, DB932A, are phenyl-heterocyclic-benzimidazoles bearing a terminal imidazoline ring at position X on the phenyl side group (Table 2). Micromolar activity in cell culture was retained if the imidazoline was moved to the corresponding terminal position (position Z) on the benzimidazole side group (DB953) but was lost if the imidazoline was present at both positions (DB302). Activity was also lost with single terminal modifications by noncyclic additions such as an amidine (at position X; DB771) or *N*-isopropylamidine (at position Z; DB954). Interestingly, activity was retained in DB compounds bearing dual terminal amidine groups (DB818A and DB293) but not if the amidines were extended by *N*-cyclopentylamidine groups (DB331).

Available for study were several phenyl-heterocyclic-benzimidazoles bearing an amidine at position Z but other modifications at position X (Table 2). Substitutions for the amidine at position X that retained micromolar activity included methoxy (DB2263) or phenyl (DB1275) groups, as well as a few examples of nonbranching extensions comprised of flexibly linked aromatic rings (DB2228, DB2229, and DB2268). In contrast, activity was lost with substitutions of amide (—CONH_2_) (DB1933 and DB1932) or nitro (DB472) groups. These findings demonstrate the critical nature of the terminal element to the micromolar activity of the pharmacophore. Previous studies with some DB compounds have demonstrated the impact of terminal imidazoline or amidine substitutions on cellular localization and DNA interaction. For example, a single amidine localizes to the cytoplasm, while dual amidines or a single imidazole localize to the nucleus (28), and both side groups facilitate interactions necessary for DNA binding (e.g., hydrogen bonding) and intercalation (33, 34, 36, 38).

The presence of a series of simply linked aromatic ring structures—the central element of the pharmacophore—was a feature common to these DB library compounds. Evidence suggests that a benzimidazole side group to this element, at least when terminally modified by an amidine (e.g., DB293 [Table 2]), provides a structural motif important to micromolar activity and which cannot be substituted with a phenyl ring (DB075 [Table 4]). Consistent with this interpretation is the retention of micromolar activity when a benzimidazole is added back as a side group (DB2214 [Table 4]) or when this benzimidazole side group is substituted with the similar dual ring structure of quinoline (e.g., DB818A versus DB1504 [Table 1]).

With regard to the central structure of this core element, a comparison of bis-benzimidazole compounds (Table 3) shows that micromolar activity is associated with single 5- or 6-membered aromatic ring structures but not if expanded by linkage with additional aromatic rings (DB2147 and DB2137) or the presence of a noncyclic linkage between central phenyl groups (DB2265). This suggests a critical molecular length to the core element of the pharmacophore. Given these findings among the bis-benzimidazoles and the characteristics of diaryl-heterocyclic compounds with micromolar activity, it would appear that the core element is more commonly comprised of three or four simply linked single or dual planar aromatic ring structures. The core element of many of the selected DB compounds included a 5-membered heterocyclic aromatic ring. Comparing otherwise equivalent phenyl-heterocyclic-benzimidazoles indicates that a central thiophene ring confers modest improvement in micromolar antiprion activity and potency (i.e., lower TC-EC_50_) over a central furan ring (Table 2) (DB932A > DB772, *P*_Bon_ = 0.005). A similar trend was seen among the bis-benzimidazoles, where the greatest micromolar activity and improved potency were associated with a central thiophene (DB1033), a selenophene (DB1310), or a methylpyrrole ring (DB191 versus DB192); a furan bis-benzimidazole was not available for comparison. This suggests the importance of the furan/thiophene/selenophene moiety to the core element of the pharmacophore. For example, DNA binding studies with some DB compounds have demonstrated that the inclusion of a central ring and additional aromatic rings (e.g., phenyl, quinolines, and/or benzimidazoles) within the core element of the compound impacts curvature, planarity, and length, all of which affect positioning for DNA interaction (33, 35).

Several compounds of structural interest were also selected to probe the potential molecular mechanism of antiprion action. Specifically, we examined the effects of these compounds on the presumed primary mechanism of prion replication (i.e., PrP^Sc^-seeded misfolding activity as measured by sPMCA assay) and on the stability of misfolded PrP accumulating during disease as measured by PK resistance. Interference with either of these processes is anticipated to reduce prion infectivity, and recently, stabilization of misfolded PrP has been associated with polythiophene-induced reduction in prion infectivity.

DB772, DB932A, and DB818A inhibited PrP^Sc^-seeded misfolding during the sPMCA assay (Fig. 5 and 6) but failed to affect PK resistance at 1 μM, suggesting that the PrP^Sc^-seeded misfolding reaction is a target of these DB compounds and that PrP^Sc^ stabilization may not be the mechanism of inhibition (Fig. 7). Interestingly, DB948 had potent cellular and stabilization activities; however, it failed to inhibit PrP^Sc^-seeded misfolding. DB771 failed to affect not only PrP^Sc^ accumulation in cell culture but also that of PrP^Sc^-seeded misfolding and PK resistance. As discussed above, DB771 contains a single structural difference from DB772, the substitution of the imidazoline with an amidine, suggesting that a single imidazoline is favored over a single amidine in the inhibition of PrP^Sc^-seeded misfolding. DB302 also lacked micromolar activity in cell culture and significantly impacted cell viability (Table 2); however, DB302 inhibited PrP^Sc^-seeded misfolding (Fig. 6B). This activity reinforces the role of an imidazoline at position X and suggests that the substitution at the Z locus of DB302 potentially altered the cellular target or compound metabolism, resulting in off-target effects.

In conclusion, the present study has further evaluated the antiprion activity of DB772 and identified 11 additional compounds displaying potent inhibitory activity in a cell line derived from a natural TSE host. The data suggest that the target for this inhibition is likely through PrP^C^ misfolding to PrP^Sc^. Further, a SAR analysis has revealed key molecular structures associated with inhibition. A more complete characterization of the preliminary hit compounds is required, including *in vivo* testing and an examination of pharmacokinetic properties such as stability, solubility, and blood-brain barrier permeability. To precisely determine a structural basis for antiprion activity, a deeper investigation into the activities of untested compounds from the DB compound library must also be performed. The identification of structures contributing to PrP^Sc^ inhibition will further aid in the elucidation of mechanisms underlying prion pathogenesis and in the development of highly effective TSE therapeutics.

## Supplementary Material

Supplemental material
