# Supplementary material for: Antiprion Activity of DB772 and Related Monothiophene- and Furan-Based Analogs in a Persistently Infected Ovine Microglia Culture System
Source: Antimicrob Agents Chemother. 2016 Aug 22;60(9):5467–82. doi: 10.1128/AAC.00811-16 (PMC4997874; doi:10.1128/AAC.00811-16)
Supplement: Supplemental material [file supp_60_9_5467__index.html]

Antiprion Activity of DB772 and Related Monothiophene- and Furan-Based Analogs in a Persistently Infected Ovine Microglia Culture System — Supplemental material 

# Antiprion Activity of DB772 and Related Monothiophene- and Furan-Based Analogs in a Persistently Infected Ovine Microglia Culture System

## Supplemental material

- Supplemental file 1 -

  Figures S1 and S2 and Tables S1-S3

  PDF, 1.5M
