# Supplementary material for: Antiprion Activity of DB772 and Related Monothiophene- and Furan-Based Analogs in a Persistently Infected Ovine Microglia Culture System
Source: Antimicrob Agents Chemother. 2016 Aug 22;60(9):5467–82. doi: 10.1128/AAC.00811-16 (PMC4997874; doi:10.1128/AAC.00811-16)
Supplement: Supplemental material [file AAC.00811-16_zac009165497so1.pdf]

Supplemental Materials

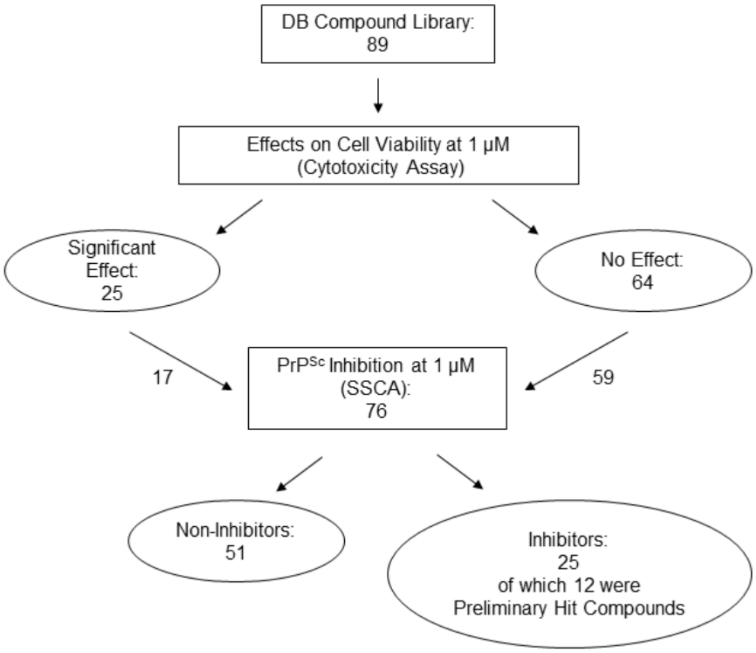

FIG S1 Flowchart of DB compound screen and selection.

TABLE S1 DB Compound Library

Compound	Structure
DB075	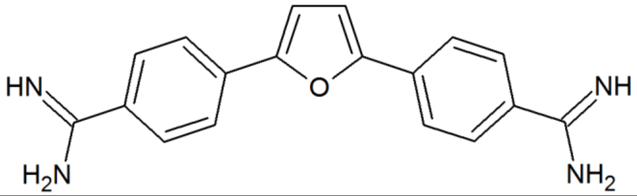
DB191	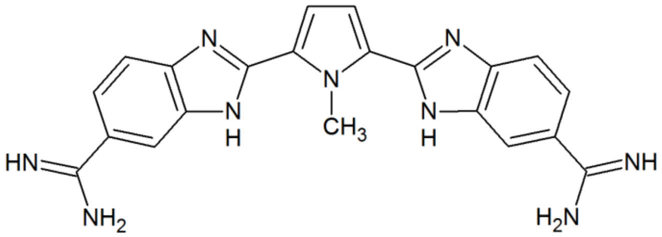
DB192	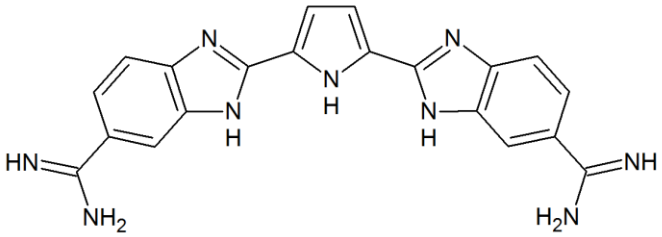
DB205	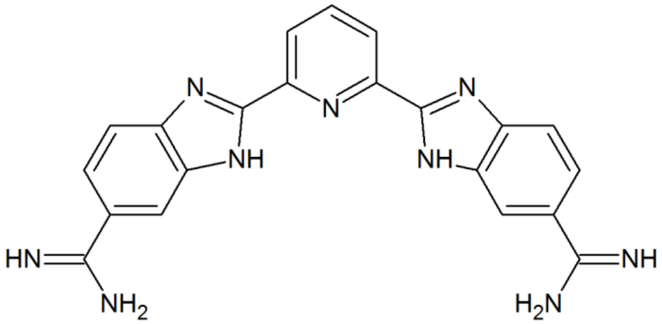
DB293	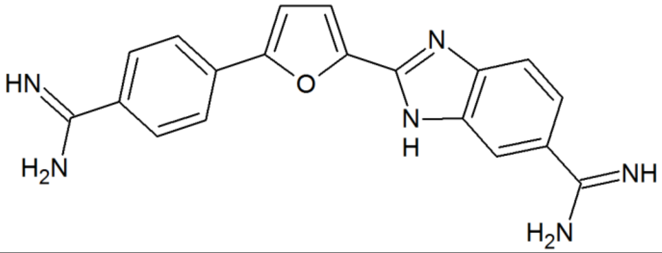
DB302	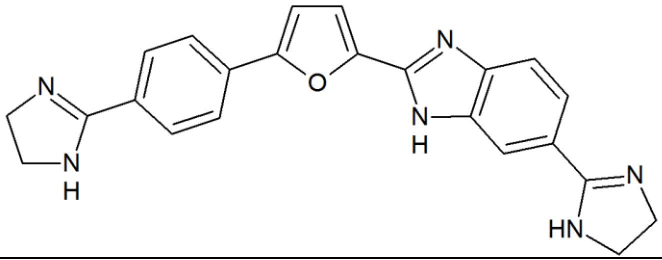

DB331	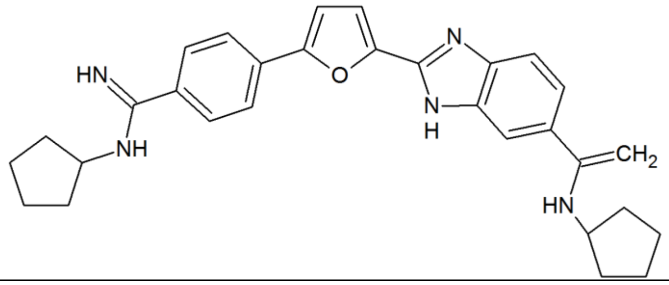
DB456	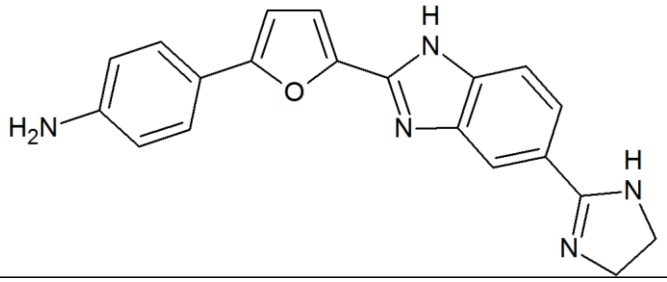
DB457	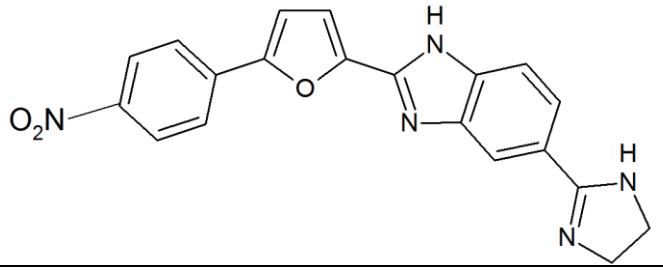
DB472	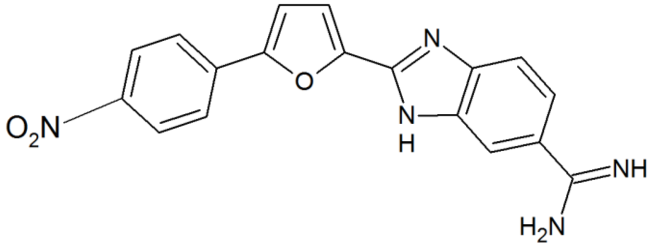
DB756	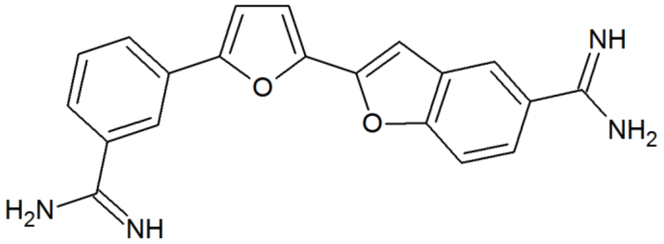
DB771	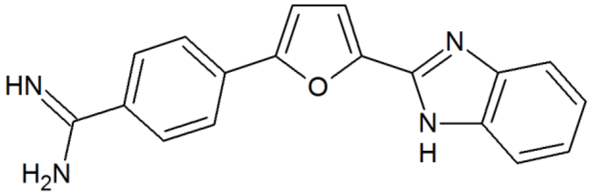
DB772	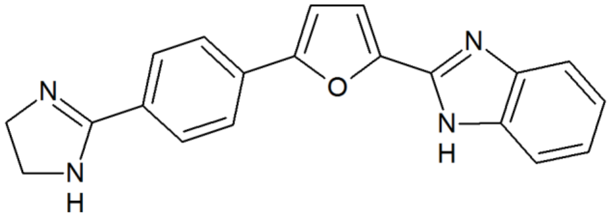

DB787	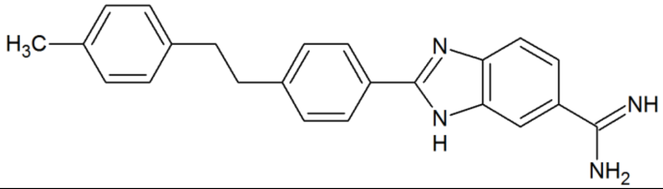
DB795	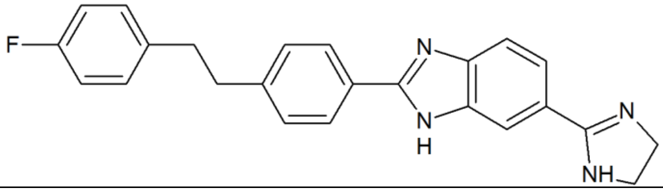
DB796	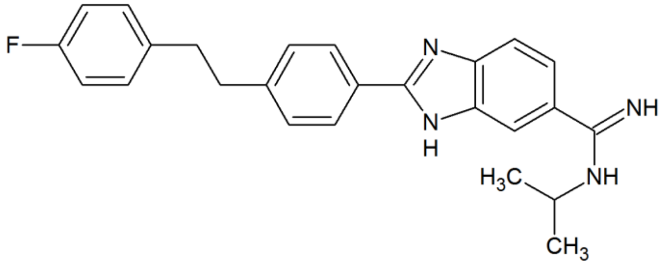
DB809A	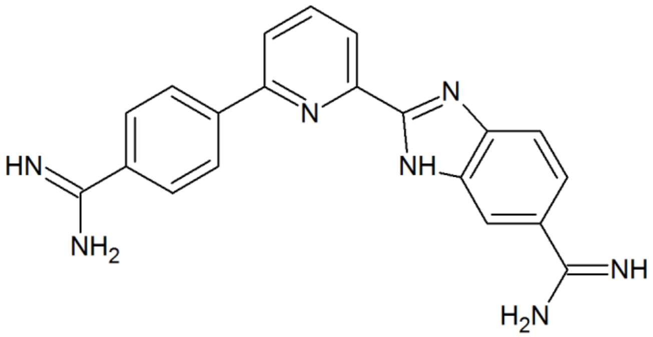
DB818A	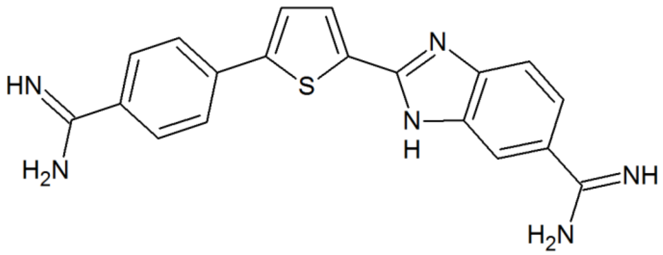
DB828	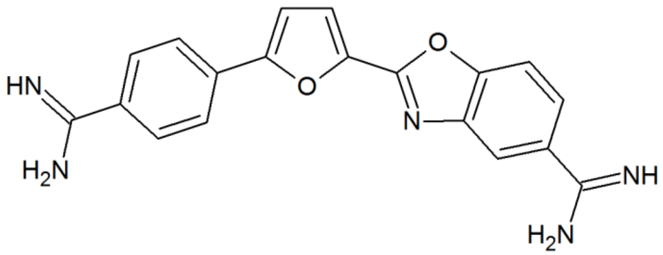
DB850	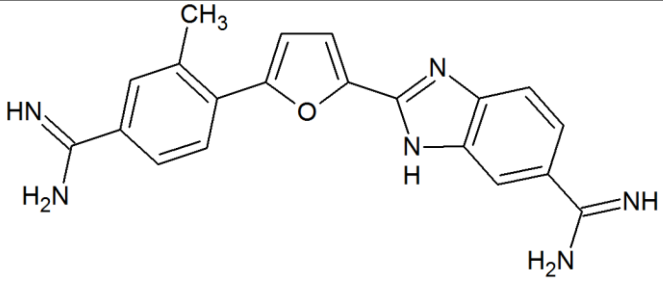

DB875	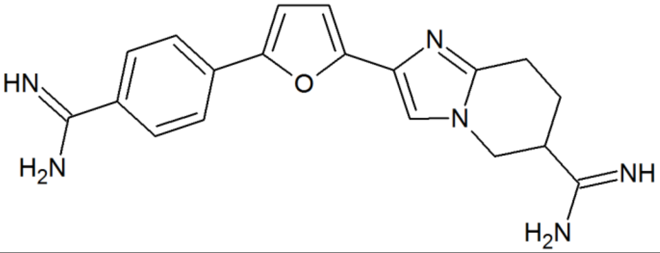
DB877A	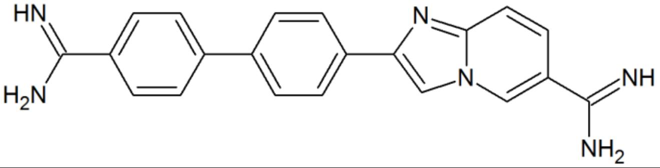
DB900	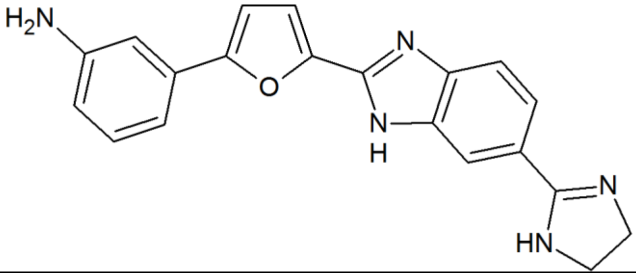
DB915	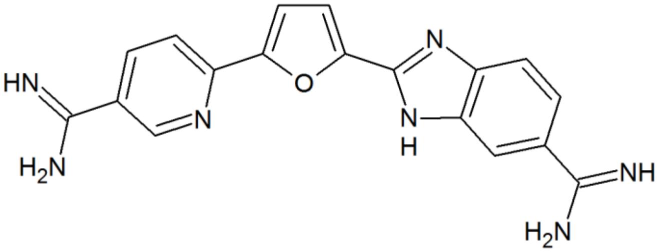
DB920	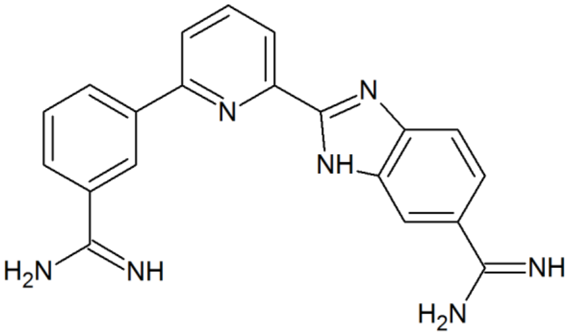
DB921A	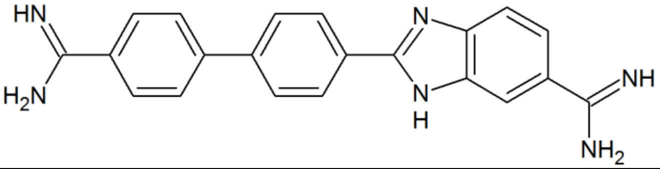
DB927	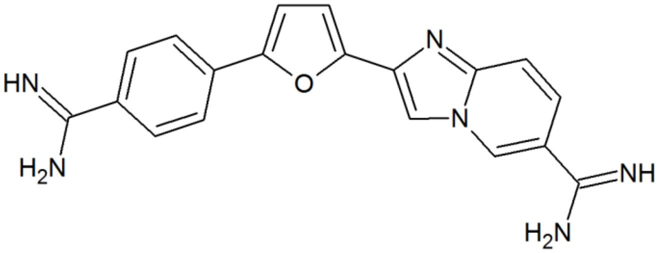

DB928	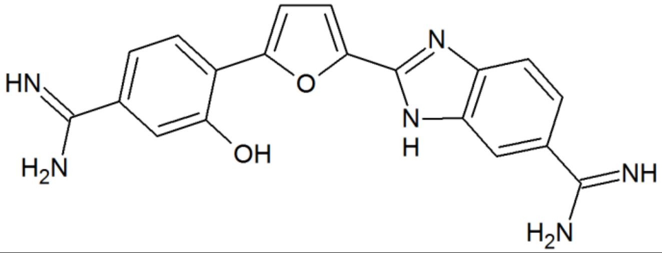
DB932A	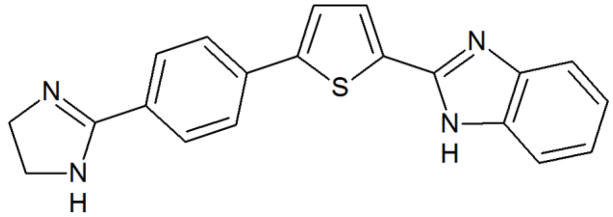
DB939	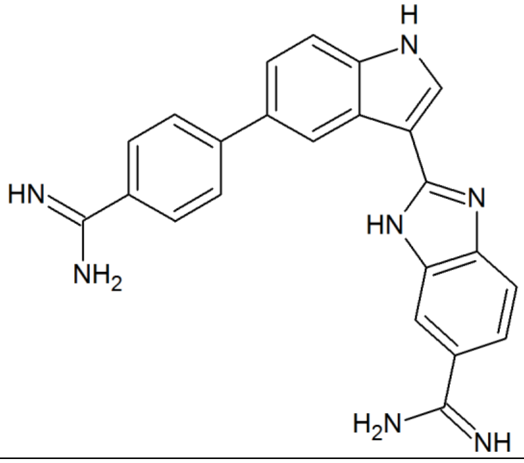
DB948	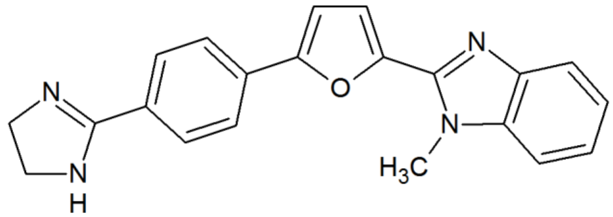
DB953	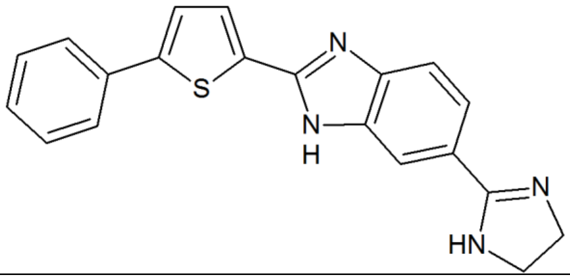
DB954	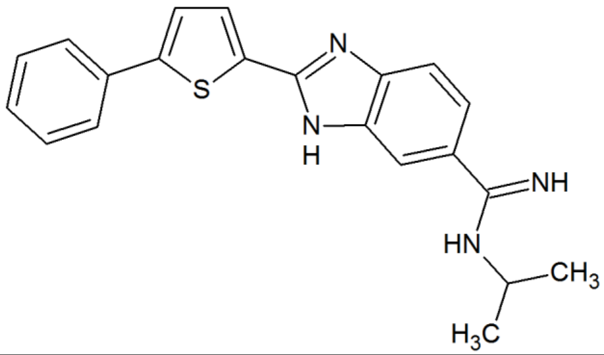

DB960	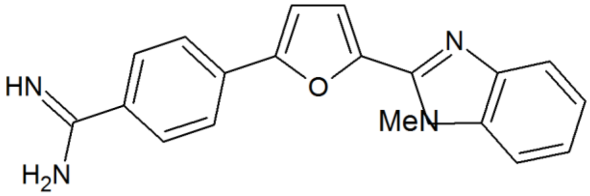
DB963	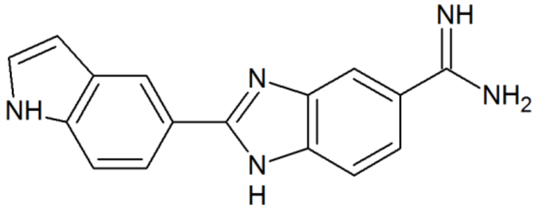
DB985	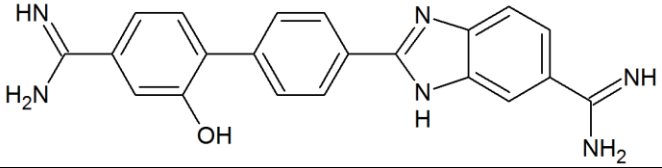
DB988	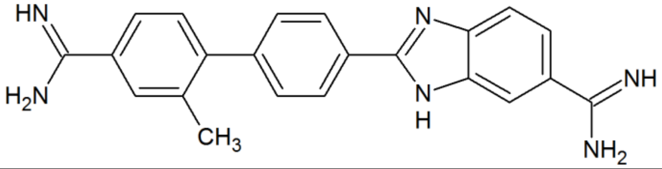
DB989	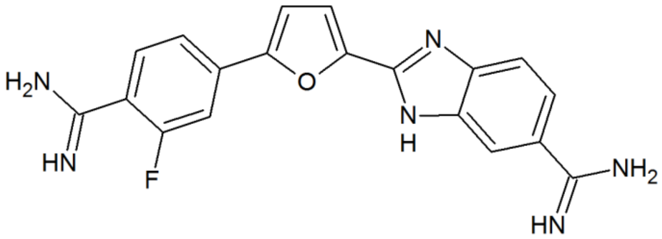
DB992	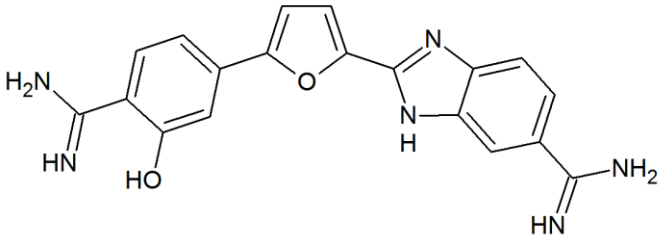
DB1013	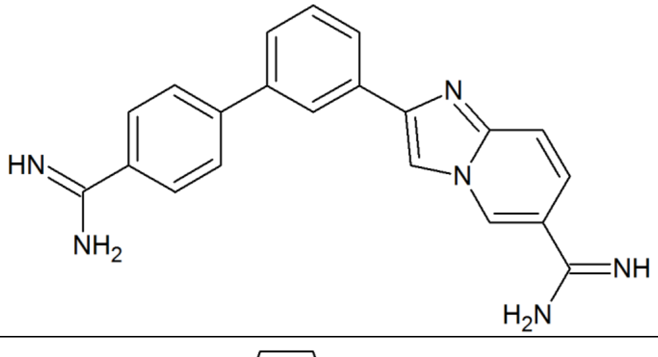
DB1030	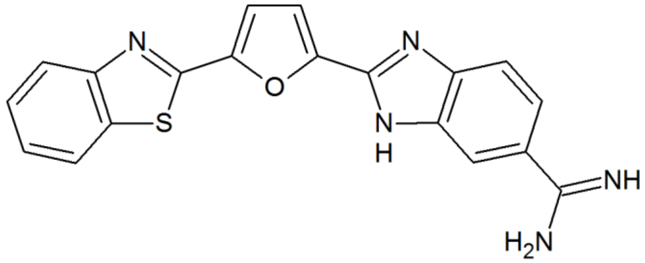

DB1031	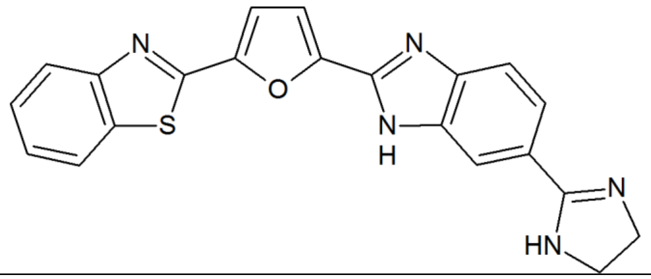
DB1033	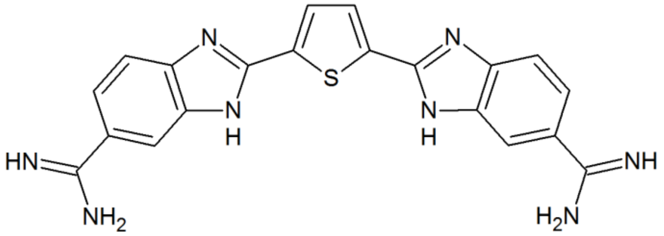
DB1034	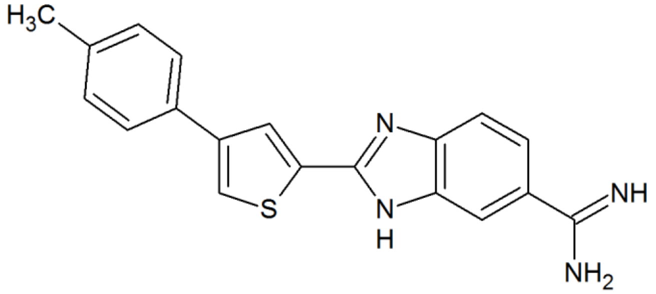
DB1055d	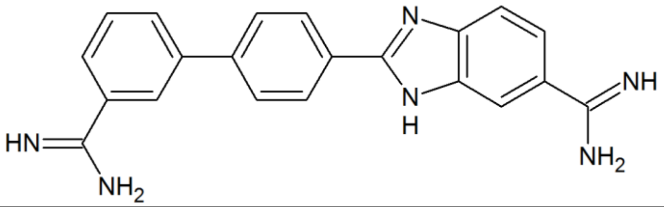
DB1100	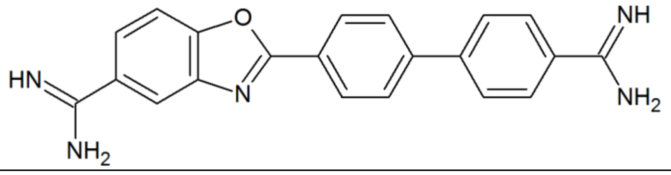
DB1114	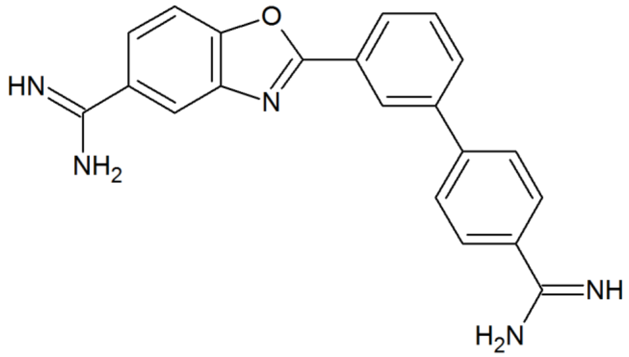

DB1119	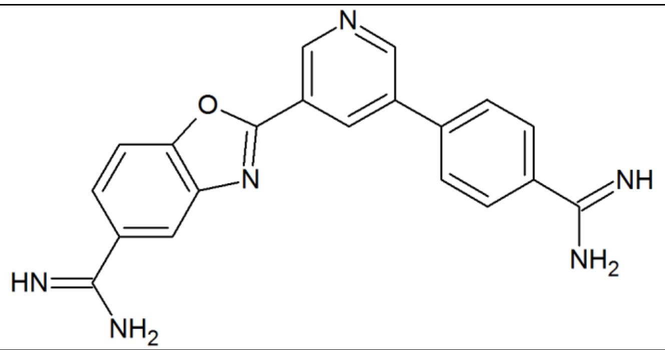
DB1173	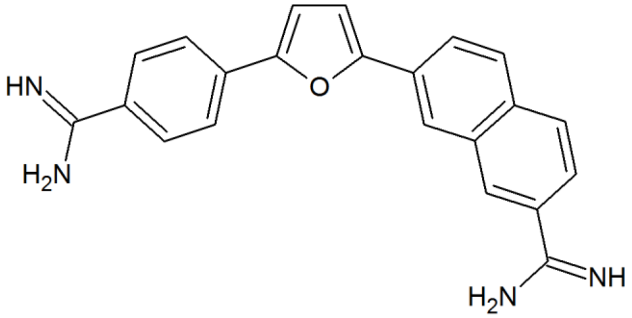
DB1175	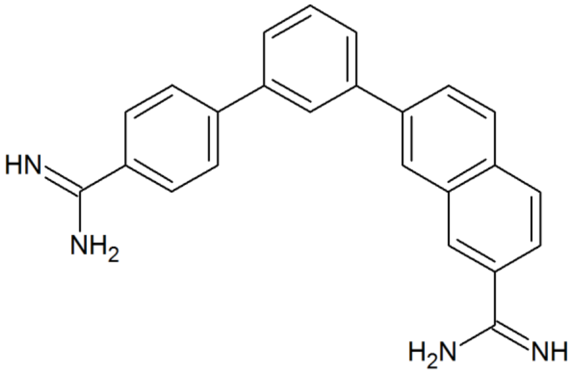
DB1177	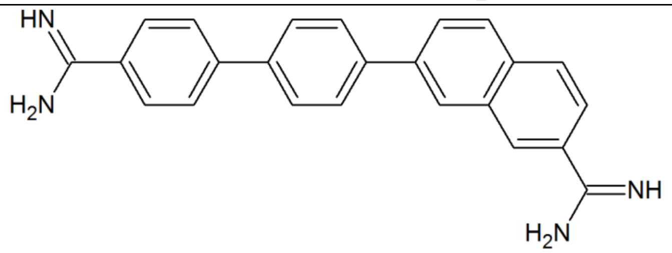
DB1208	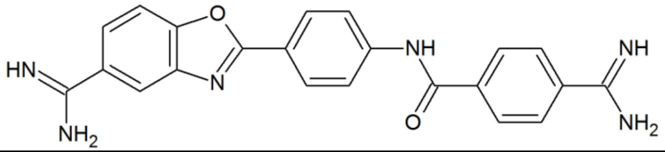
DB1256	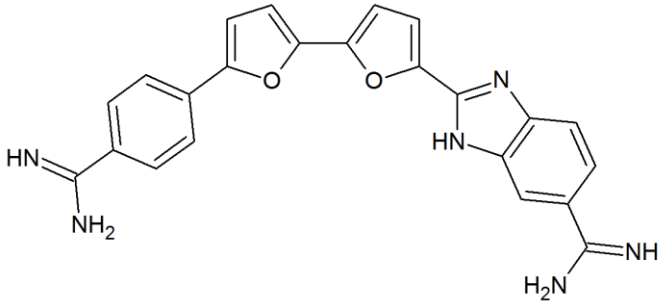

DB1264	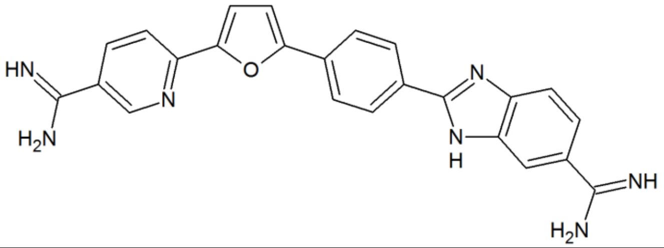
DB1275	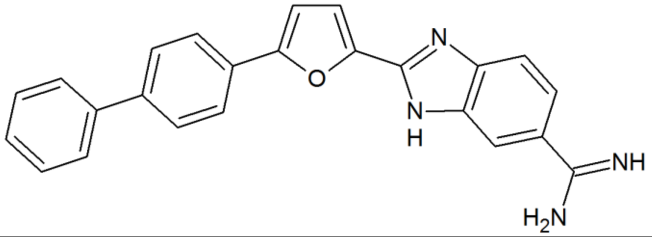
DB1310	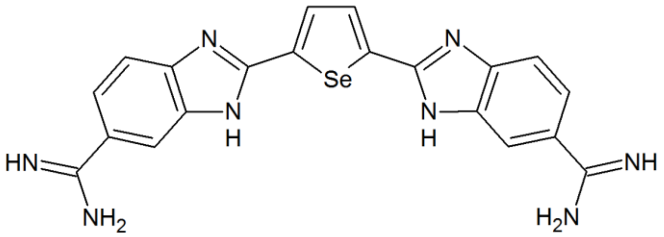
DB1344	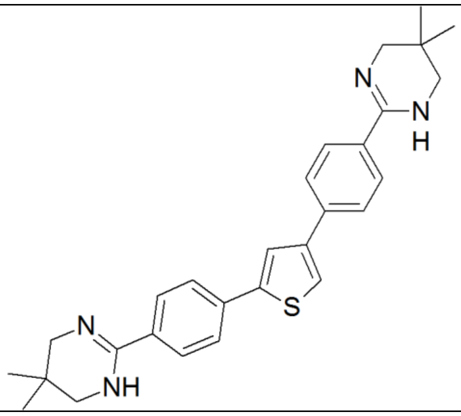
DB1478	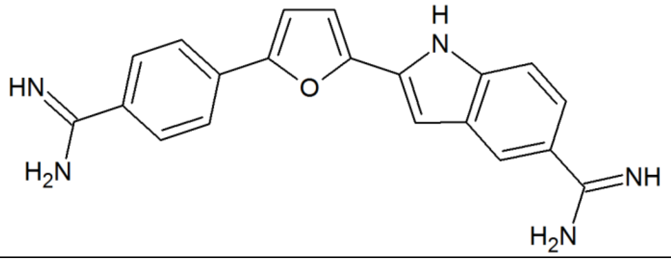
DB1481	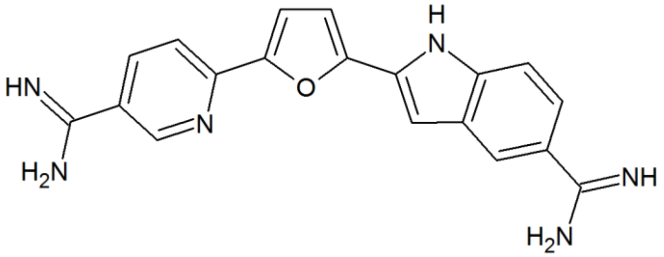

DB1504	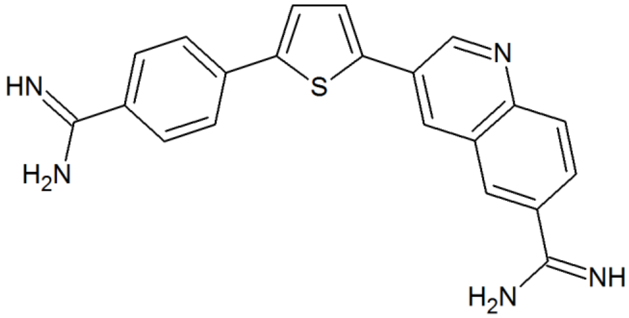
DB1601	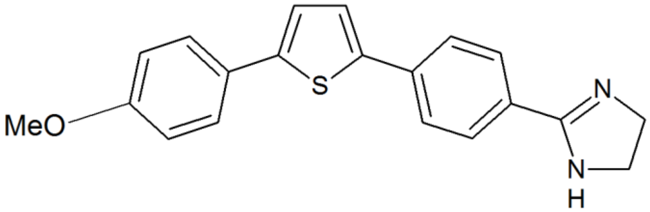
DB1770	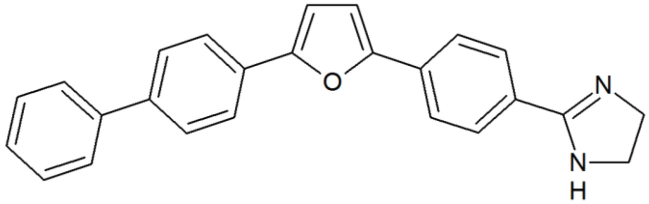
DB1798	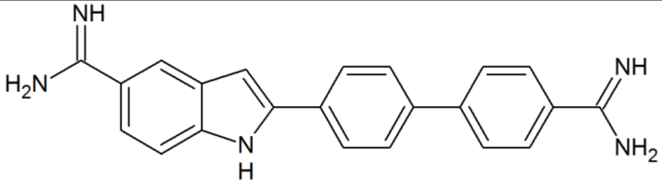
DB1804	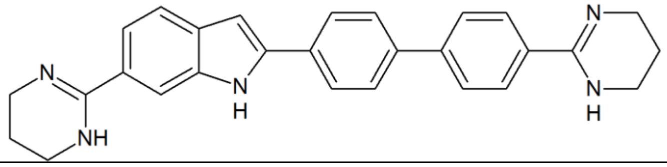
DB1879	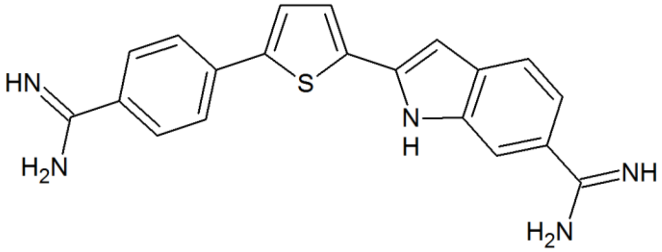
DB1894	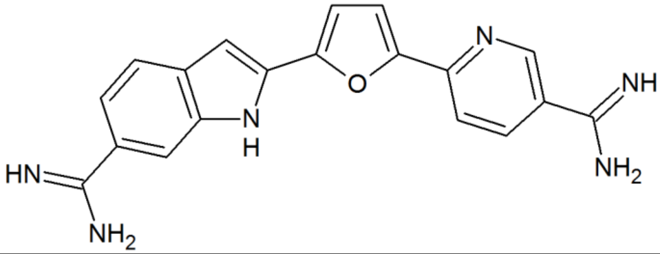
DB1896	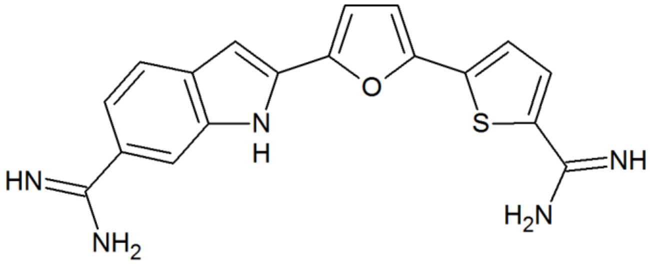

DB1932	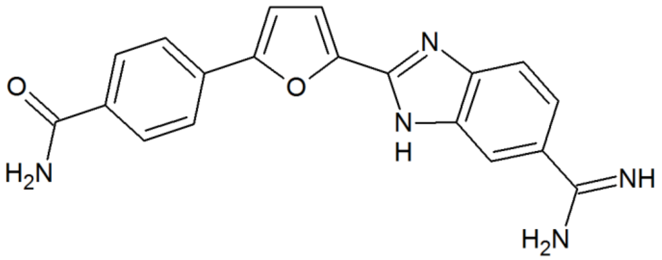
DB1933	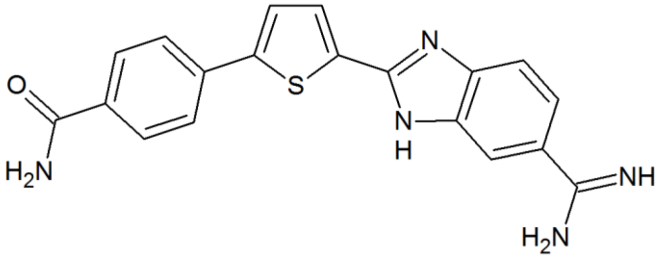
DB1963	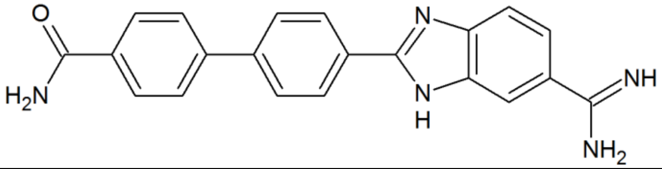
DB1970	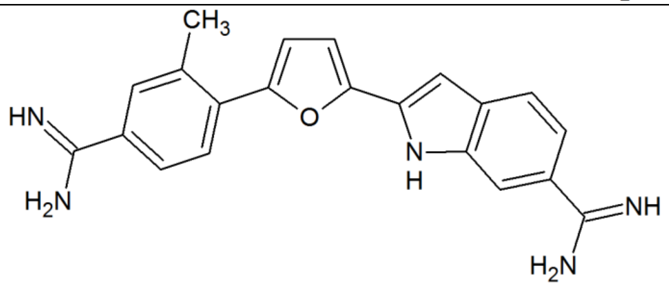
DB2002A	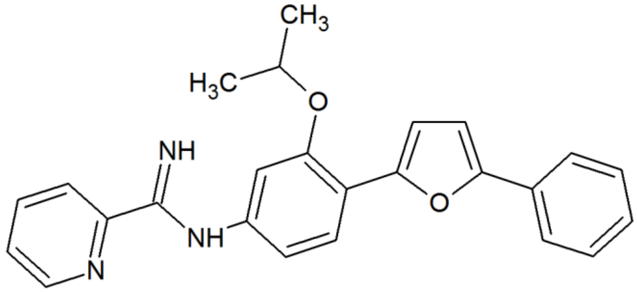
DB2005	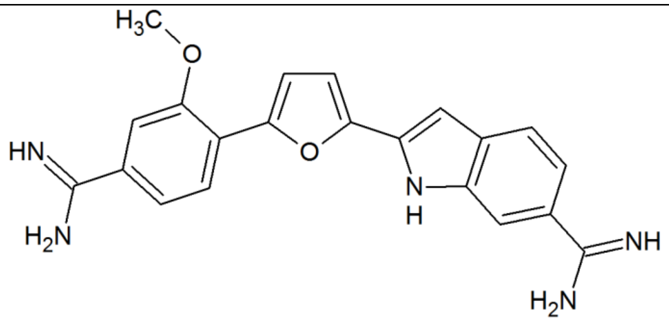

DB2009	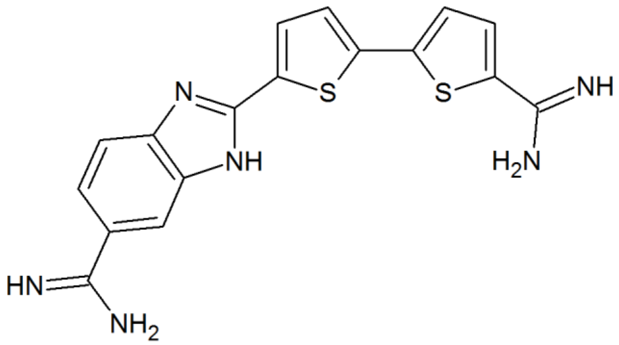
DB2033	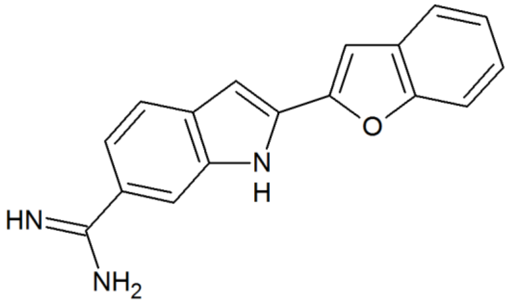
DB2073	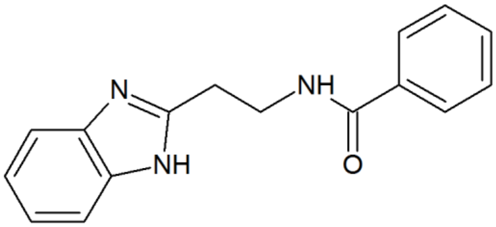
DB2090	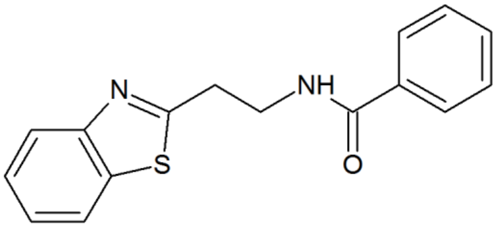
DB2104	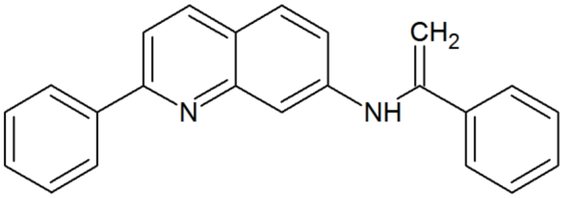
DB2137	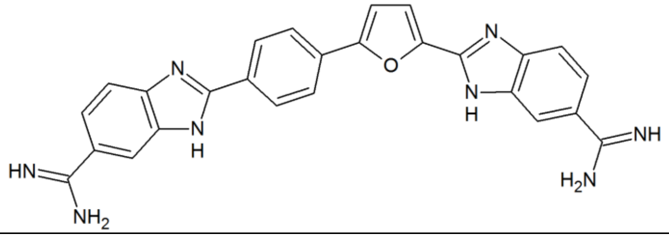

DB2147	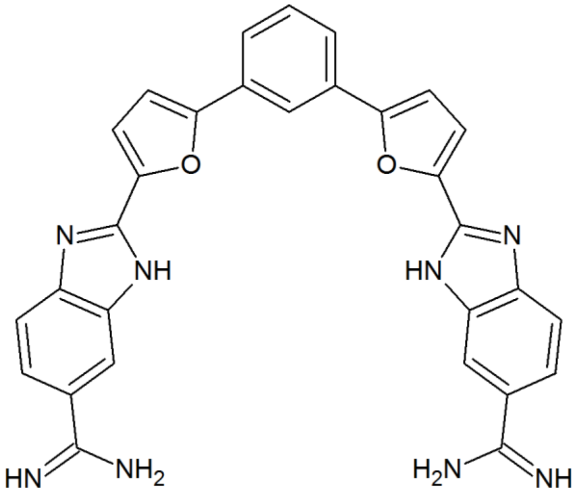
DB2195	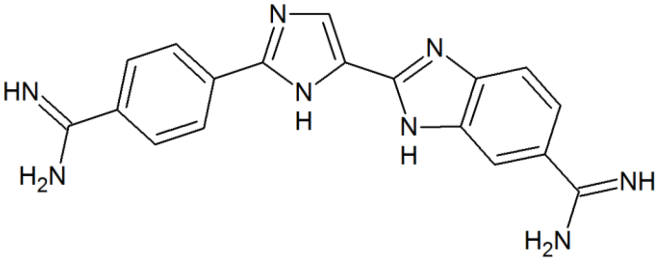
DB2196	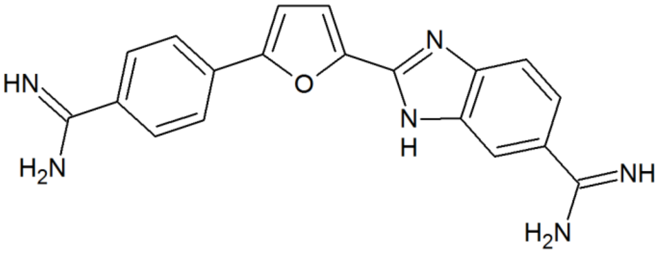
DB2214	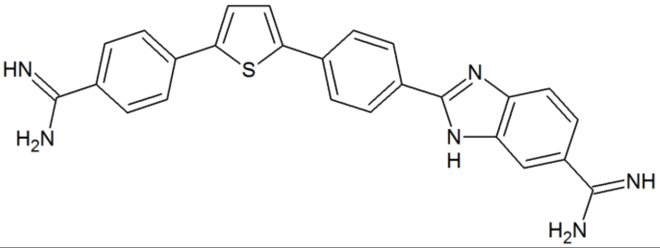
DB2223	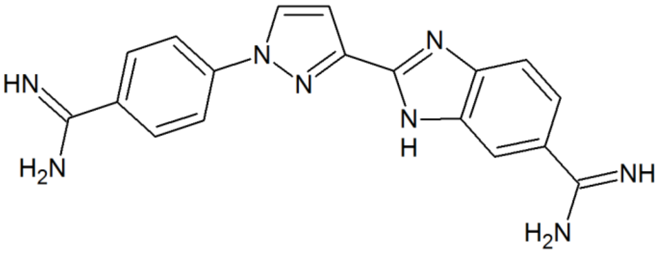
DB2228	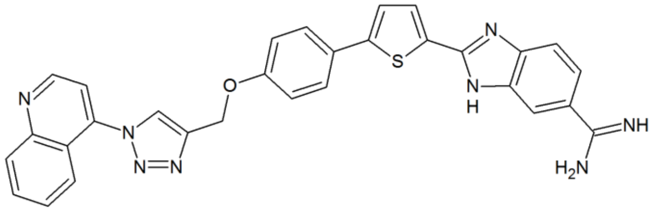

DB2229	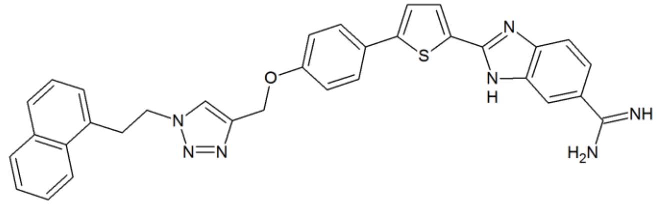
DB2263	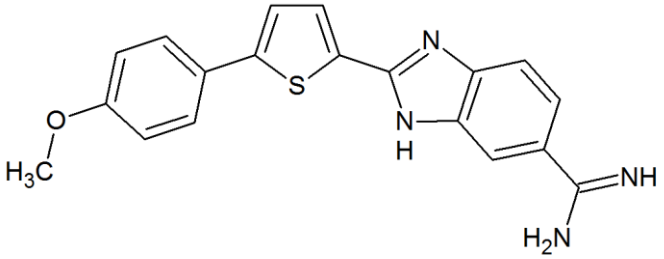
DB2265	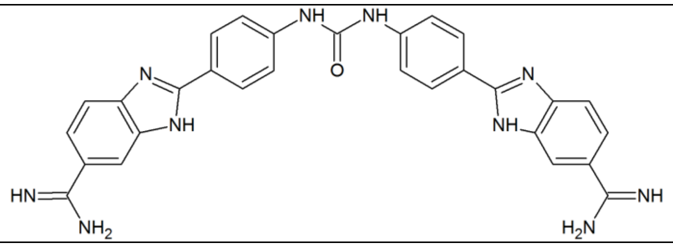
DB2268	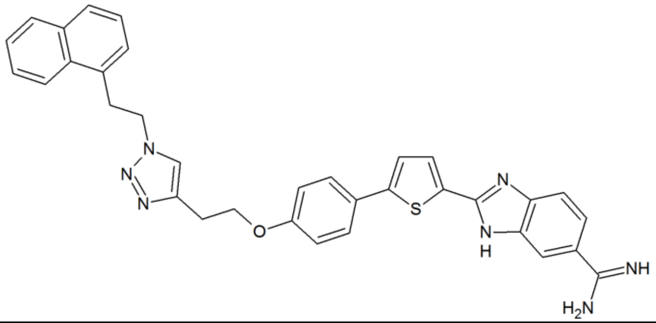

TABLE S2 Comparison of Cell-Based Effects of Preliminary Hit DB Compounds

Compound[a]	Activity at Day 4[b,c]			
	% Reduction in PrP^{Sc}[d]	% Cell Count[e]	% Viability[d]	% BrdU Incorporation[f]
DMSO	0	100	100	100
DB948	83[g]	68[g]	60[g]	Not tested
DB1033	81[g]	96	91	68[g]
DB1310	77[g]	92	110	91
DB818A	74[g]	85	170	76[g]
DB932A	70[g]	92	81	66[g]
DB2214	69[g]	84	180[g]	68[g]
DB1264	68[g]	89	138	77
DB772	66[g]	89	96	78
DB191	65[g]	88	188[g]	74[g]
DB2228	63[g]	82	120	92
DB192	61[g]	98	150[g]	101
DB1504	57[g]	102	156	100

[a]Preliminary hit DB compounds ordered by decreasing PrP^{Sc} inhibitory activity at 1 μM.

[b]Activity relative to vehicle control on day 4 of 1 μM treatment.

[c]Average activity from at least 3 independent experiments.

[d]Values from SSCA or WST-1 assay shown in Fig. 1.

[e]Values from trypan blue staining and counting.

[f]Values from BrdU assay shown in Fig. 2.

[g]Significant effect compared to vehicle control (P_{Dun}<0.05).

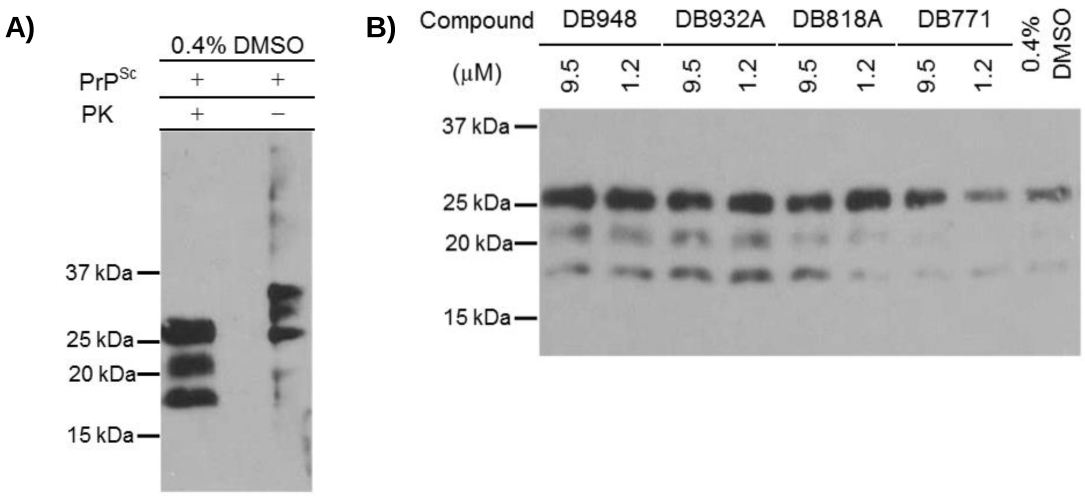

FIG S2 The effects of selected DB compounds on PrP resistance to PK-digestion. (A) Shown are the three PrP glycoforms present in ScBH (PrP^{Sc}+), itself a mixture of PrP^{C} and PrP^{Sc}, before (–) and after (+) PK digestion. Note the lack of any full length or intermediate sized PrP bands after PK digestion. (B) Shown is a representative immunoblot from the experiment on the effect of selected DB compound pre-treatments of ScBH on PK digestion (summarized data presented in Fig. 7B). Again, note the lack of full length or intermediate sized PrP bands after PK digestion.

TABLE S3 Extended Compounds

Ext.	Compound	Structure at Position X
Ext 1	DB2228	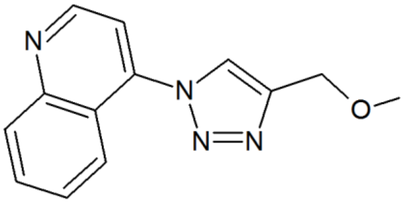
Ext 2	DB2229	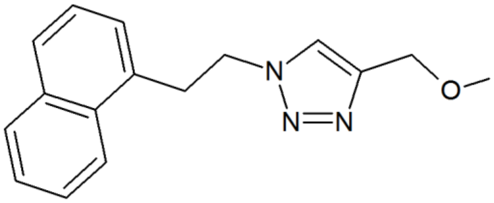
Ext 3	DB2268	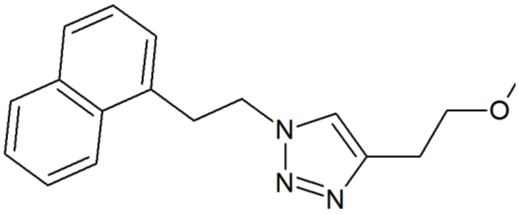
